# The eIF2α kinase HRI triggers the autophagic clearance of cytosolic protein aggregates

**DOI:** 10.1074/jbc.RA120.014415

**Published:** 2020-12-02

**Authors:** Tapas Mukherjee, Valeria Ramaglia, Mena Abdel-Nour, Athanasia A. Bianchi, Jessica Tsalikis, Hien N. Chau, Suneil K. Kalia, Lorraine V. Kalia, Jane-Jane Chen, Damien Arnoult, Jennifer L. Gommerman, Dana J. Philpott, Stephen E. Girardin

**Affiliations:** 1 Department of Laboratory Medicine and Pathobiology, University of Toronto, Toronto, Ontario, Canada; 2 Department of Immunology, University of Toronto, Toronto, Ontario, Canada; 3 Krembil Research Institute, Toronto Western Hospital, University Health Network, Toronto, Canada; 4 Institute of Medical Engineering & Science, MIT, Cambridge, Massachusetts, USA; 5 INSERM U1197, Hôpital Paul Brousse, Bâtiment Lavoisier, Villejuif Cedex, France; 6 Université Paris-Saclay, Paris, France

**Keywords:** HRI, integrated stress response, alpha-synuclein, Parkinson’s disease, protein aggregation, proteasome, autophagy, protein misfolding, BAG3–HSPB8 complex, CASA, ATF4, activating transcription factor 4, BAG3, B-cell lymphoma-2 associated athanogene 3, CASA, chaperone-assisted selective autophagy, cUPR, cytosolic unfolded protein response, HRI, heme-regulated inhibitory, HSP, heat shock protein, HSPB8, heat shock protein 8, ISR, integrated stress response, SC, scramble sequence, shHRI, small hairpin RNA directed against HRI sequence

## Abstract

Large cytosolic protein aggregates are removed by two main cellular processes, autophagy and the ubiquitin-proteasome system, and defective clearance of these protein aggregates results in proteotoxicity and cell death. Recently, we found that the eIF2α kinase heme-regulated inhibitory (HRI) induced a cytosolic unfolded protein response to prevent aggregation of innate immune signalosomes, but whether HRI acts as a general sensor of proteotoxicity in the cytosol remains unclear. Here we show that HRI controls autophagy to clear cytosolic protein aggregates when the ubiquitin-proteasome system is inhibited. We further report that silencing the expression of HRI resulted in decreased levels of BAG3 and HSPB8, two proteins involved in chaperone-assisted selective autophagy, suggesting that HRI may control proteostasis in the cytosol at least in part through chaperone-assisted selective autophagy. Moreover, knocking down the expression of HRI resulted in cytotoxic accumulation of overexpressed α-synuclein, a protein known to aggregate in Parkinson’s disease, dementia with Lewy bodies, and multiple system atrophy. In agreement with these data, protein aggregate accumulation and microglia activation were observed in the spinal cord white matter of 7-month-old *Hri*^−/−^ mice as compared with *Hri*^*+/+*^ littermates. Moreover, aged *Hri*^−/−^ mice showed accumulation of misfolded α-synuclein in the lateral collateral pathway, a region of the sacral spinal cord horn that receives visceral sensory afferents from the bladder and distal colon, a pathological feature common to α-synucleinopathies in humans. Together, these results suggest that HRI contributes to a general cytosolic unfolded protein response that could be leveraged to bolster the clearance of cytotoxic protein aggregates.

Protein misfolding and aggregation are at the heart of neurodegenerative processes and can occur extracellularly as well as in the cytosol, the latter being evidenced by reported aggregation of various cytosolic proteins, including α-synuclein (Parkinson’s disease and other synucleinopathies), tau (Alzheimer’s disease and tauopathies), and TDP-43 (amyotrophic lateral sclerosis and frontotemporal dementia) (1). Central to the field of neurodegeneration is the idea that avoiding accumulation of these protein aggregates could prevent disease development and progression. Recent efforts have focused on understanding the mechanisms underlying the dynamic clearance of these aggregates, and in particular the role of autophagy and proteasome-mediated protein turnover in this process (2, 3). However, how cells actually detect the initial formation of these aggregates to trigger their clearance is surprisingly poorly understood. Filling this gap would, most likely, greatly contribute to the design of novel therapeutic strategies in brain diseases. Indeed, understanding the underlying mechanisms accounting for the accumulation of, and the cell-to-cell propagation of, proteotoxic fibrillar aggregates represents a major theme in multiple neurodegenerative diseases.

Heme-regulated kinase inhibitor (HRI), or eIF2α kinase 1, is one of the four eIF2α kinases, along with PERK, GCN2, and PKR, which collectively respond to a variety of cellular stresses and thereby trigger the integrated stress response (ISR) (4). The main characteristics of the ISR are (i) the induction of a global translation shutoff directly caused by the phosphorylation of the translation initiation factor eIF2α and (ii) the upregulation of a stress-associated transcriptional reprograming dependent in part on the transcription factors ATF4 and ATF3. HRI was initially identified as a factor essential for controlling the expression of globin in red blood cells in response to changing levels of heme through a mechanism involving the dissociation of the chaperones Hsp90 and Hsc70 (5, 6). However, HRI is ubiquitously expressed and induces eIF2α phosphorylation in various cell types in response to an array of stresses. In particular, the role of HRI in controlling the ISR in response to oxidative stress is well characterized (7, 8). Indeed, phosphorylation of eIF2α and accumulation of eIF2α-dependent mRNA stress granules in cells treated with sodium arsenite, a potent oxidative stress inducer, is fully HRI dependent. Whether HRI responds directly to the oxidative environment or to the consequences of the oxidative stress, such as accumulation of misfolded proteins, is currently unclear. However, the latter scenario is more likely given that HRI is also responsible for controlling eIF2α phosphorylation in response to heat shock (9) and proteasome inhibition (10), two conditions that lead to the accumulation of misfolded proteins in the cytosol.

In line with the proposed role of HRI in favoring cellular responses to proteotoxic stress and to the accumulation of misfolded proteins in the cytosol, we have recently demonstrated that the rapid engagement of several families of intracellular sensors of microbes, which are known to assemble into large molecular platforms (or signalosomes), require active control by an HRI-dependent pathway (11). This regulatory pathway, dependent on HRI, the heat shock protein HSPB8, eIF2α, ATF4 and ATF3, was coined the cytosolic unfolded protein response (cUPR), as it shares similarities with the pathway of regulation of protein folding in endoplasmic reticulum known as the unfolded protein response. In the absence of the cUPR, antimicrobial signalosomes are misfolded and display a defective capacity to trigger innate immune responses, such as proinflammatory signaling. This led to the more general suggestion that the cUPR in general, and HRI-dependent signaling in particular, could contribute to the homeostatic regulation of proteostasis in the cytosol, in part by inhibiting protein translation (following eIF2α phosphorylation) and by triggering a stress-dependent ATF3- and ATF4-dependent transcriptional reprogramming in response to the accumulation of cytosolic misfolded proteins.

In the context of neurodegenerative disorders, for which accumulation of cytotoxic protein aggregates is thought to play a major role, the potential contribution of HRI-dependent stress signaling remains uncharacterized. Interestingly, rare mutations in *EIF2AK1* and the related gene *EIF2AK2* (which encodes the eIF2α kinase PKR) are associated with developmental delay, white matter alterations, cognitive impairment, and movement disorders (12). These observations are in line with previous reports that mutations in *EIF2B* are also associated with similar manifestations (13) and, more generally, that eIF2α is critical for neuronal health by integrating cellular stress pathways of the ISR (14). In agreement, it was recently observed that neuronal expression of HRI, although very low in resting conditions, was significantly increased following inhibition of protein degradation using a proteasome inhibitor, which resulted in constitutive inhibition of new protein synthesis through the HRI-eIF2α axis (15). This suggests that HRI plays a key role in neurons to maintain proteostasis, which could prevent accumulation of misfolded and potentially toxic proteins in these cells. Here, we provide evidence that HRI is critical for cytoprotection against proteotoxicity in cellular models of proteasome inhibition, likely by enhancing the autophagic degradation of protein cargos. Moreover, overexpression of α-synuclein, a protein known to be cleared by autophagy-mediated processes accumulated and was cytotoxic in HRI-silenced cells. *In vivo*, aged *Hri*^−*/*−^ mice displayed accumulation of protein aggregates and phospho-S129 α-synuclein in the central nervous system, supporting the notion that HRI-dependent cUPR may represent a novel mechanism that could be targeted to enhance clearance of protein aggregates in the context of neurodegenerative diseases.

## Results

### HRI controls aggresome formation in response to proteasome inhibition

**Figure 1:**
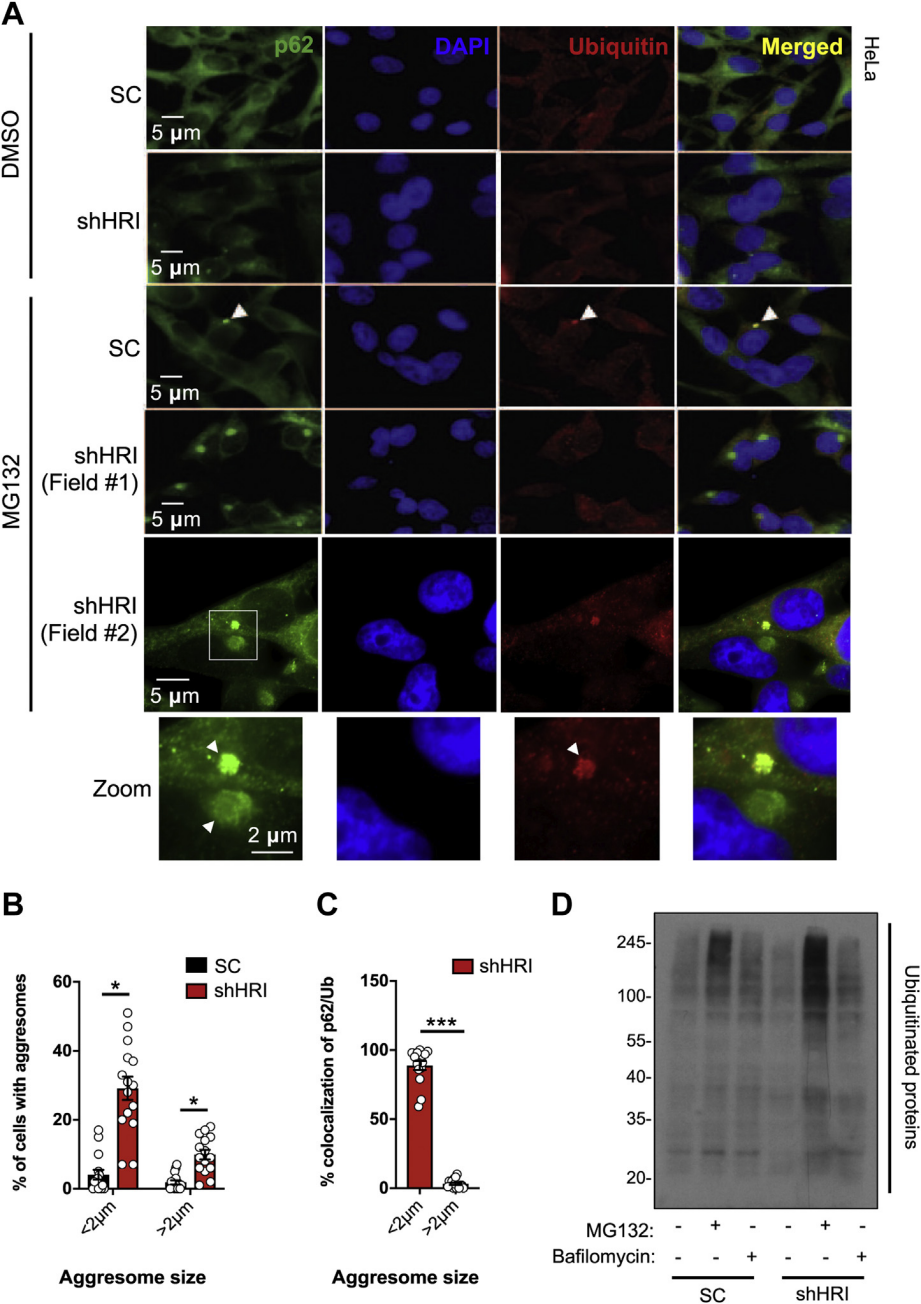
**HRI controls aggresome formation in response to proteasome inhibition.***A*–*C*, HeLa cells transduced with lentiviral particles, targeting either a scrambled (SC) sequence or HRI (shHRI), were treated with either DMSO (control) or 5 μM MG132 for 4 h and stained with anti-p62 and anti-ubiquitin antibodies and analyzed by immunofluorescence (*A*) (the scale bar represents 5 or 2 μm, as indicated) and quantified for the percentage of cells that have aggresomes larger and smaller than 2 μm (*B*) and for the frequency that these aggresomes have p62 and ubiquitin colocalization (*C*). *D*, HEK293T cells transduced with lentiviral particles targeting a scrambled sequence (SC) and HRI (shHRI) and treated for 4 h with 10 μM MG132 and 10 nM bafilomycin and Western blot analysis was carried out using antibodies against ubiquitinated proteins. *A* and *D* are representative of three independent experiments. Bar graphs in B and C represent means ± SEM of aggresomes quantified from five images each from the three independent experiments indicated in *A*. ∗ and ∗∗∗ represent *p* < 0.05 and 0.001, respectively.

We silenced the expression of HRI in HeLa cells using small hairpin RNA directed against HRI sequence (shHRI) or a scramble sequence (SC) (Fig. S1*A*). Cells were then treated for 4 h with 5 μM MG132, a proteasome inhibitor, and fixed cells were analyzed by immunofluorescence using antibodies against p62 and ubiquitin. As previously reported, proteasome inhibition using MG132 resulted in accumulation of p62+ and ubiquitin+ foci in a cytosolic area adjacent to the nucleus, known as the aggresome (16), in a small percentage of cells (Fig. 1*A*). Aggresomes result from the accumulation, at the microtubule organizing center, of proteins cargos that are destined for lysosomal degradation and migrated in a centripetal way along the microtubule network located near the perinuclear region. Stimulation of the cells with higher doses of MG132 (up to 100 μM) and for longer periods of time (up to 16 h) resulted in increased frequency and size of aggresomes, but this was associated with substantial cell death (data not shown). In HRI-silenced cells, we observed that both the frequency and architecture of MG132-induced aggresomes were dramatically affected. Aggresomes occurred more frequently in HRI knockdown cells (Fig. 1, *A*–*B*) and also displayed larger diameter in average (1.84 ± 0.24 μm in HRI-silenced cells *versus* 1.35 ± 0.14 μm in control cells). Interestingly, although most small size aggresomes in control and HRI-silenced cells were both p62+ and ubiquitin+, large aggresomes (>2 μm in diameter), which were found nearly exclusively in HRI-silenced cells, were p62+/ubiquitin- (see examples of large aggregates in Field #1, Fig. 1*A* and quantifications in Fig. 1*C*). Moreover, proteasome inhibition, but not inhibition of autophagy using bafilomycin treatment, resulted in the accumulation of ubiquitinated proteins in HRI-silenced HEK293T cells, as observed by Western blotting on whole cellular extracts (Fig. 1*D* and Fig. S1*B*), suggesting that HRI silencing is in epistasis with autophagy rather than the UPS pathways.

Together, these results suggest that, during cellular stress induced by the inhibition of the proteasome, HRI plays a critical role in controlling the dynamic targeting and flux of protein aggregates that are destined for degradation. In proteasome-inhibited cells, the absence of HRI resulted in the accumulation of aggresomes, the net buildup of ubiquitin-positive proteins in cells, and the formation of atypical and very large aggresomes that were p62+/ubiquitin−.

### HRI controls proteotoxicity in the face of proteasome inhibition through modulation of autophagy

**Figure 2:**
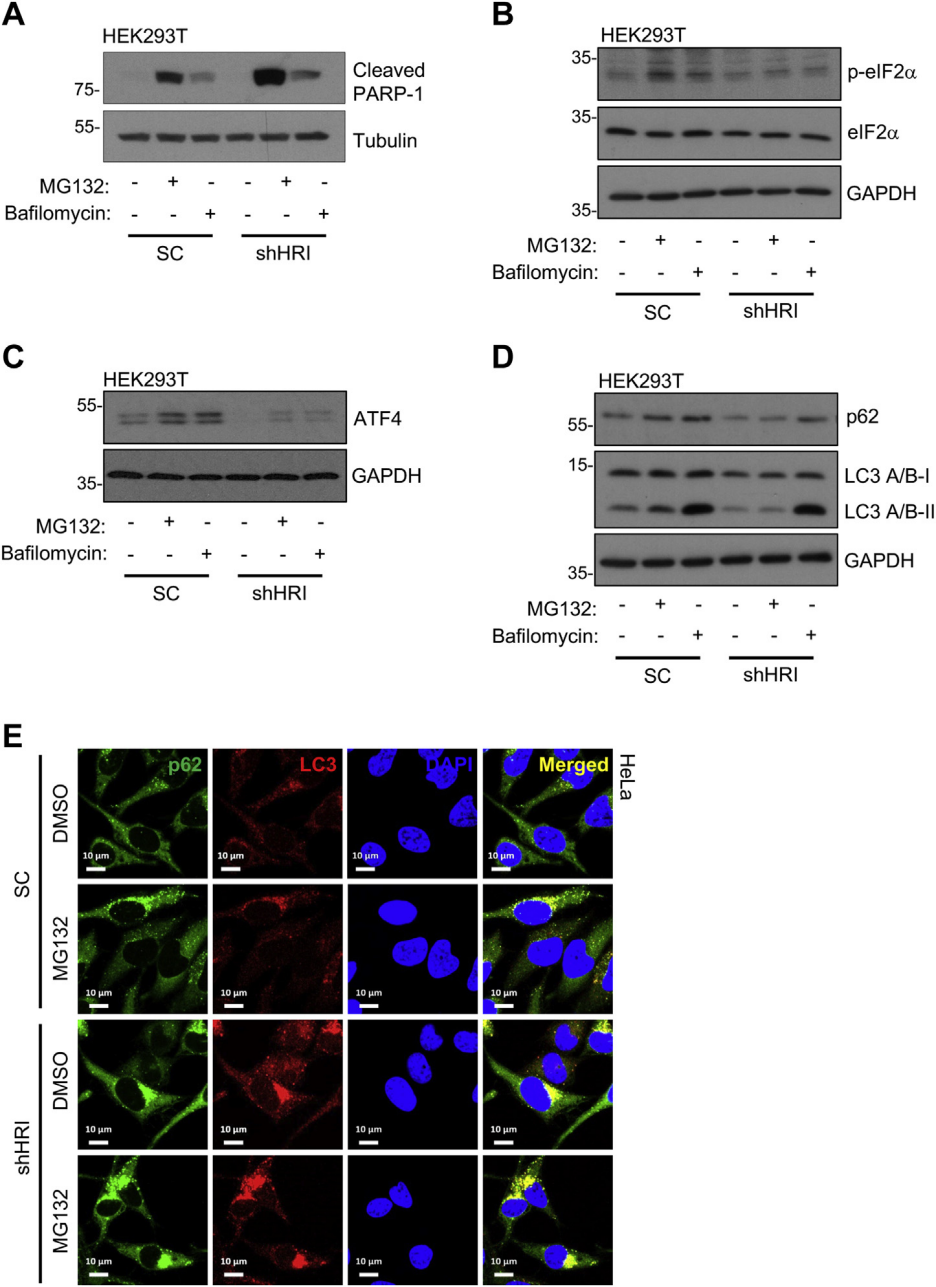
**HRI controls proteotoxicity in the face of proteasome inhibition through modulation of autophagy.***A*–*D*, HEK293T cells transduced with lentiviral particles targeting a scrambled sequence (SC) and HRI (shHRI) were treated for 4 h with DMSO (control), 10 μM MG132, and 10 nM bafilomycin, and cell extracts were collected for Western blot analysis. Western blotting was performed using antibodies against phospho-eIF2α (ser 51) or eIF2α (*A*), ATF4 (*B*), LC3 A/B or p62 (*C*), and cleaved PARP-1 (*D*). GAPDH and Tubulin were used as loading controls. *E*, scrambled (SC) sequence and HRI (shHRI) HeLa cells treated either with DMSO (CTR) or with 5 μM MG132 for 4 h was followed by fixation with ice-cold 100% methanol for 5 min. Subsequently, immunofluorescence analysis was executed using anti-p62 and anti-LC3 antibodies. *A*–*D* and *E* are representative of four and three independent experiments, respectively. The scale bar represents 10 μm.

Proteotoxicity and cell death are common features of the uncontrolled accumulation of protein aggregates. Treatment of HEK293T cells with 10 μM MG132 for 4 h resulted in apoptotic cell death, as observed using Western blotting by the accumulation of the cleaved form of PARP-1, and this cell death was exacerbated in shHRI cells (Fig. 2*A*). This effect was not caused by a general increase of sensitivity of shHRI cells to cell death because scramble-transduced and HRI-silenced cells were equally sensitive to cell death induced by tumor necrosis factor plus cycloheximide (Fig. S2*A*). Similarly, CRISPR-Cas9–mediated HRI knockout cells were more sensitive to MG132 treatment compared with wildtype (WT) HEK293T cells (Fig. S2, *B*–*C*). Furthermore, we also noticed an upregulation of the protein CHOP, a marker of cellular stress typically associated with cell death, in shHRI cells upon treatment with MG132 in comparison with SC cells (Fig. S2*D*). Importantly, shHRI cells appeared to be equally sensitive to autophagy inhibition by bafilomycin as control cells (Fig. 2*A*), thus showing that HRI silencing does not lead to a general sensitivity to the defective clearance of cytosolic cargos. Again, these results argue for the fact that HRI silencing is epistatic with autophagy pathways, which explains why HRI-silenced cells are particularly sensitive to the inhibition of the other arm of cytosolic protein clearance pathways, the UPS.

Upon inhibition of UPS by proteasomal inhibitors, HRI was shown to phosphorylate eIF2α (10, 17). In addition, activating transcription factor 4 (ATF4), which is induced downstream of eIF2α phosphorylation, triggers autophagy during proteosomal inhibition (18, 19). As eIF2α-ATF4 pathway has been previously proposed to regulate stress or infection-induced expression of autophagy pathway genes (18, 20), we first questioned if HRI engages the eIF2α-ATF4 signaling axis when the UPS is blocked. In support of this, we observed impaired phosphorylation of eIF2α in shHRI cells upon treatment with 20 μM MG132 in comparison with SC cells (Fig. 2*B*). Similar results were also obtained with 10 nM bafilomycin, which inhibits autophagosome and lysosome fusion (Fig. 2*B*), although the effects were more modest. Interestingly, we found that basal ATF4 protein and transcript levels were significantly reduced in shHRI cells (Fig. 2*C* and Fig. S2*D*). Upon MG132 or bafilomycin treatment, we observed an increase in ATF4 protein levels in both SC and shHRI cells, although less so in shHRI cells (Fig. 2*C*). Furthermore, mRNA analysis revealed that ATF4 protein accumulation in either SC or shHRI cells was independent of MG132 or bafilomycin-mediated transcriptional activation (Fig. S2*D*), hence indicating that the reduced accumulation of ATF4 in shHRI cells following inhibition of the UPS or autophagic clearance was likely a consequence of blunted phosphorylation of eIF2α and the subsequent translational regulation of ATF4. Moreover, increased ATF4 protein expression in SC cells upon bafilomycin treatment was associated with an increased expression of the ATF4-target autophagy gene *SQSTM1*, encoding p62 protein, which was not observed upon MG132 treatment, and this transcriptional induction was not detected in shHRI cells (Fig. S2*F*), possibly in line with the poor induction of ATF4 in those cells. In support, at the protein level, we observed decreased cellular p62 levels in shHRI cells compared with SC cells, not only at baseline but also following treatment with MG132 or bafilomycin (Fig. 2*D*). This suggests that the HRI-ATF4 axis may directly impact on the expression of p62, a key protein involved in autophagic clearance of protein cargos, when proteostasis is compromised.

In order to analyze more directly the impact of HRI on autophagy in cells undergoing UPS inhibition, we next analyzed microtubule-associated protein light chain 3 (LC3) by Western blot, as conversion of nonlipidated LC3 (LC3-I) to lipidated LC3 (LC3-II) is a marker of autophagy (21) and LC3-II migrates faster than LC3-I in Western blot. Interestingly, treatment with 20 μM MG132 led to an increase in the lipidated form LC3-II in SC but not shHRI cells, directly supporting that autophagy induction was blunted in shHRI cells when the UPS was blocked. In contrast, stimulation with 10 nM bafilomycin that prevents autophagolysosome formation and thereby, protein turnover, resulted in similar levels of accumulation of LC3-II in both SC and shHRI cells (Fig. 2*D*), suggesting that autophagy as a whole was not compromised in shHRI cells when the UPS was functional but that the effect was only revealed when the bulk of protein turnover normally assigned to the UPS was offloaded to the autophagic machinery. This observation is consistent with our observation that cytotoxicity induced by bafilomycin was similar in SC and shHRI cells (see above Fig. 2*A*). As a control, we noted that LC3 transcript levels were not significantly different between SC and shHRI cells (Fig. S2*F*). In line with the notion that autophagic clearance of protein cargos was compromised in shHRI cells when UPS was blocked, we finally observed in immunofluorescence that the strong p62+ perinuclear aggresomes observed in shHRI cells after MG132 treatment were also LC3+ (Fig. 2*E*), highlighting that these cargos that accumulate are indeed autophogosomes. So, taking together the fact that the total amount of LC3-II is reduced in MG132-treated shHRI cells with the observation that LC3+ autophagosomes accumulate in aggresomes in these cells, these data collectively suggest that the autophagic machinery is defective in shHRI cells and cannot properly handle the excess load of misfolded proteins resulting from UPS inhibition. This may be in part caused by the upregulation of the eIF2α-ATF4 signaling axis upon UPS inhibition, which in turn could regulate expression of autophagy genes, such as p62.

### HRI regulates the expression of BAG3 and HSPB8, two key components of chaperone-assisted selective autophagy

**Figure 3:**
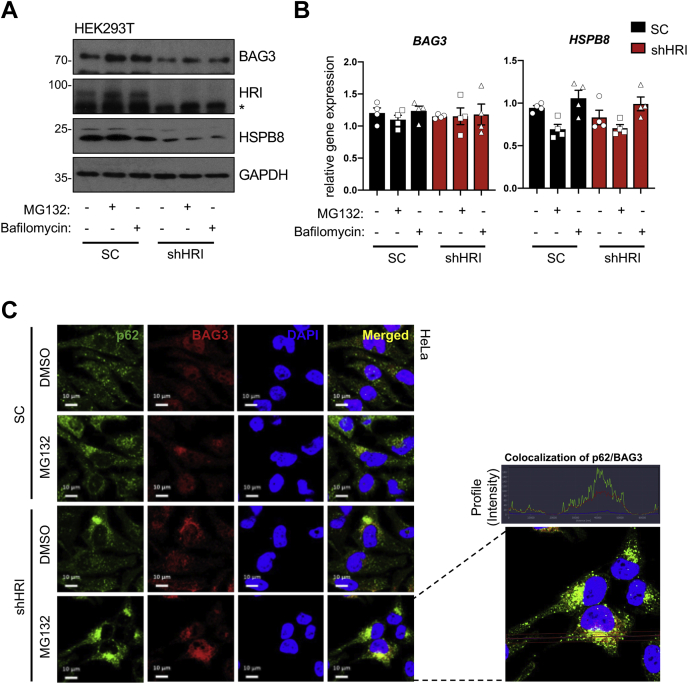
**HRI regulates BAG3 and HSPB8, two key proteins of chaperone-assisted selective autophagy, during proteotoxic stress.***A*–*B*, cell extracts from SC and shHRI HEK293T cells treated with DMSO (control), 10 μM MG132, and 10 nM bafilomycin for 4 h were collected and either Western blot analysis was performed using antibodies probing for BAG3, HRI, HSPB8, and GAPDH (loading control) (*A*) or qRT-PCR was carried out for indicated genes *(B*). *C*, scrambled (SC) sequence and HRI (shHRI) HeLa cells treated either with DMSO (control) or with 5 μM MG132 for 4 h was followed by fixation with 4% paraformaldehyde for 10 min and then were subjected to immunofluorescence analysis using anti-BAG3 and anti-p62 antibodies. Enlarged panel of MG132-treated shHRI HeLa cells on the right indicates perinuclear location of BAG3-p62+ complexes. *A*–*B* and *C* are representative of four and three independent experiments, respectively. The scale bar represents 10 μm.

Previous studies had identified that heat shock protein 8 (HSPB8) and B-cell lymphoma-2 associated athanogene 3 (BAG3) chaperone complex facilitate targeting of misfolded proteins for degradation by macroautophagy (22, 23). HSPB8 belongs to the HSPB family of molecular chaperones, whereas BAG3 is a member of the cochaperone family of Bag domain-containing proteins. Moreover, mutations in *HSPB8* and *BAG3* have been reported to be associated with neuropathy and myopathy (24, 25, 26). Furthermore, the BAG3–HSPB8 complex was suggested to play a critical role in cellular proteostasis *via* chaperone-assisted selective autophagy (CASA) (26). Importantly, our previous study demonstrated that HRI regulates HSPB8 expression during cUPR (11). Therefore, we questioned if HRI silencing impacted the BAG3-HSPB8–dependent CASA, which mediates selective degradation of misfolded proteins, upon proteosomal inhibition. Interestingly, we observed reduced protein levels of BAG3 and HSPB8 in shHRI cells compared with SC cells, both in unstimulated conditions and upon 20 μM MG132 or 10 nM bafilomycin treatment (Fig. 3*A*). In contrast, no differences were observed at the transcript level (Fig. 3*B*). We speculated that the reduced levels of these two proteins implicated in CASA in shHRI cells could contribute to the overall reduced protein clearance and aggresome formation in these cells in MG132-treated cells. In support, we observed that indeed the large aggresomes observed in MG132-treated were not only p62+ but also BAG3+ (Fig. 3*C*), suggesting that the efficient clearance of p62+/BAG3+ cargos was impaired in shHRI cells, at least in part as a result of the reduced levels of the key scaffolding proteins p62 and BAG3. In order to understand if HRI silencing also influences other autophagy proteins involved in macroautophagy, we analyzed ATG16L1, which forms a complex with two other autophagy pathway proteins, ATG5-ATG12, and plays an important role in specifying the site of LC3 lipidation for membrane biogenesis during autophagy. Unlike BAG3 or HSPB8, we did not observe any changes in the levels of ATG16L1 protein or transcript (Fig. S3, *A*–*B*). In agreement with these results, we also did not observe any changes in the accumulation of ATG16L1 at the perinuclear microtubule organizing center region in shHRI cells following MG132 treatment, whereas p62+ aggresomes did (Fig. S3*C*). Altogether, these observations show that, when HRI expression is silenced, the aggresomes that accumulate upon MG132 treatment contain the CASA protein BAG3. This suggests that the autophagy impairment in these cells is at least in part associated with defective clearance by the CASA pathway and may be caused by reduced levels of BAG3 and HSPB8 proteins in shHRI cells.

### HRI controls responses to aggregating α-synuclein

**Figure 4:**
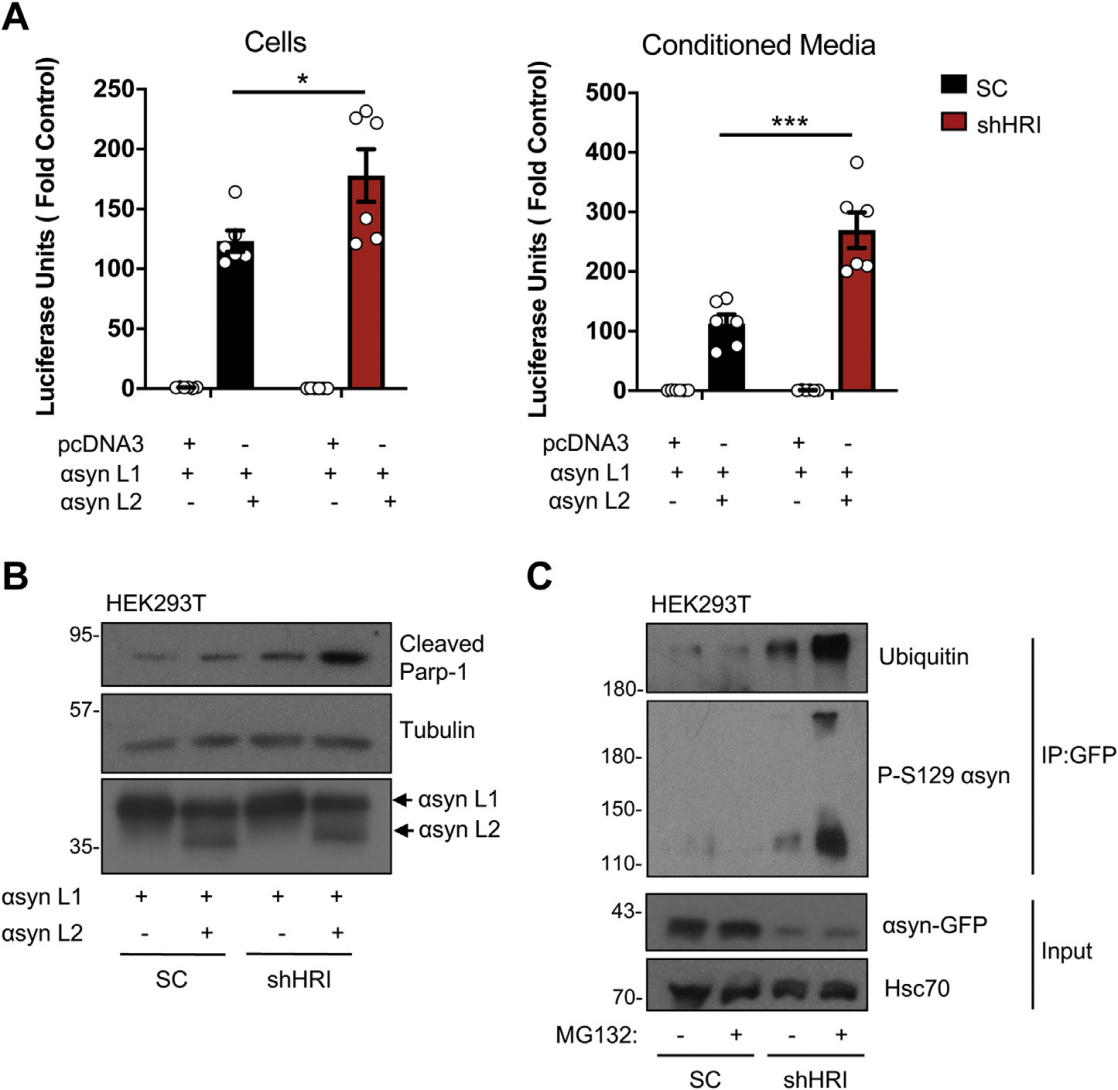
**HRI controls responses to aggregating α-synuclein.***A*, HEK293T cells transduced with lentiviral particles targeting either a scrambled sequence (SC) or HRI (shHRI) were transfected with α-synuclein fused to a truncated luciferase (αsyn L1) and either pcDNA or α-synuclein fused to the remaining luciferase fragment (αsyn L2) and luciferase assays were performed on the cells (*left*) and the conditioned media (*right*). Bar graphs display means ± SEM plotted from six independent experiments pooled from two biological sets of three each (N= 6). *B*, HEK293T cells transduced with lentiviral particles targeting either a scrambled sequence (SC) or HRI (shHRI) were transfected with αsyn L1 and either pcDNA or αsyn L2 and analyzed by Western blotting using the indicated antibodies. Coimmunoprecipitation assay using lysates of HEK293T cells transduced with lentiviral particles targeting a scrambled (SC) sequence or HRI (shHRI), transfected with GFP tagged α-synuclein following stimulation for 4 h with 5 μM MG132. ∗ and ∗∗∗ indicates *p* < 0.05 and 0.001, respectively.

α-Synuclein, a protein that accumulates and forms aggregates in neurons in Parkinson’s disease and other synucleinopathies may impair the UPS early in the disease process (27) and is dominantly cleared from the cytosol by autophagy-mediated clearance (28). To monitor the role of HRI in controlling the accumulation of α-synuclein, we used a luciferase reporter system for which a split form of the luciferase enzyme is associated as two hemi-luciferase moieties in fusion with α-synuclein (αsyn L1 and αsyn L2). As α-synuclein self-assembles, the hemi-luciferase moieties interact to generate a functional enzyme, and thus luciferase activity is a proxy of α-synuclein self-assembly (29, 30). Overexpression of αsyn L1 and αsyn L2 resulted in significantly increased luciferase activity in lysates of shHRI cells as compared with scramble control cells (Fig. 4*A*, left), suggesting that a greater self-assembly of α-synuclein occurred in HRI-silenced cells. Interestingly, the cell culture medium prior to cell lysis also displayed greater luciferase activity in shHRI cells (Fig. 4*A*, right), and the difference was actually more pronounced than in the cell lysates. We reasoned that the accumulation of functional luciferase enzyme in the cell culture medium could be caused by cell death and the release of stable aggregates containing αsyn L1/αsyn L2. In agreement with this, we observed that overexpression of αsyn L1 and αsyn L2 was sufficient to cause apoptotic cell death, and this was exacerbated in HRI-silenced cells (Fig. 4*B*), suggesting that α-synuclein overexpression is particularly cytotoxic to shHRI cells. Finally, in HRI-silenced cells, overexpression of αsyn-GFP caused accumulation of multimeric forms of α-synuclein that were phosphorylated on Ser129 (Fig. 4*C*), which is a biochemical hallmark of α-synuclein aggregation (31). Moreover, these α-synuclein multimers were ubiquitinated, suggesting that the proteins are part of cargos that are destined for degradation, which build up in the cytosol of HRI-silenced cells (Fig. 4*C*). Interestingly, the accumulation of phospho-S129 and ubiquitin-positive GFP-αsyn was strongly enhanced after inhibition of the proteasome, but only in shHRI cells (Fig. 4*C*). We interpret these data to suggest that MG132-mediated inhibition of the proteasome imposes a stress on the autophagic machinery by shifting the normal homeostatic load of cargos needing to be cleared from the UPS to autophagy; as a consequence, the overload may not be fully absorbed by the autophagic machinery in HRI-silenced cells, as autophagy functions below capacity in these cells (see Figs. 2 and 3), resulting in accumulation of phospho-S129 and ubiquitin-positive GFP-αsyn. Finally, it is worth noting that we consistently observed lower levels of overexpressed αsyn in the lysates of shHRI cells as compared with scramble control cells (see αsyn-GFP “input” Western blot, Fig. 4*C*). We believe that this is caused by the fact that α-synuclein spontaneously aggregates in HRI-silenced cells and thus disappears from the soluble fraction of our lysates. We consistently noted that greater amounts of α-synuclein could be retrieved in the radioimmunoprecipitation assay (RIPA)-insoluble fraction (solubilized in urea) of shHRI cells and that these forms of α-synuclein could only be blotted using an antibody against phospho-S129 synuclein, suggesting that the epitope recognized by the anti-α-synuclein was masked (data not shown). In sum, these results suggest that HRI plays a key role in controlling the clearance and the cytotoxicity of α-synuclein, in cellular models of protein overexpression.

### Accumulation of protein aggregates in the central nervous system of aged *Hri*^*−/−*^ mice

**Figure 5:**
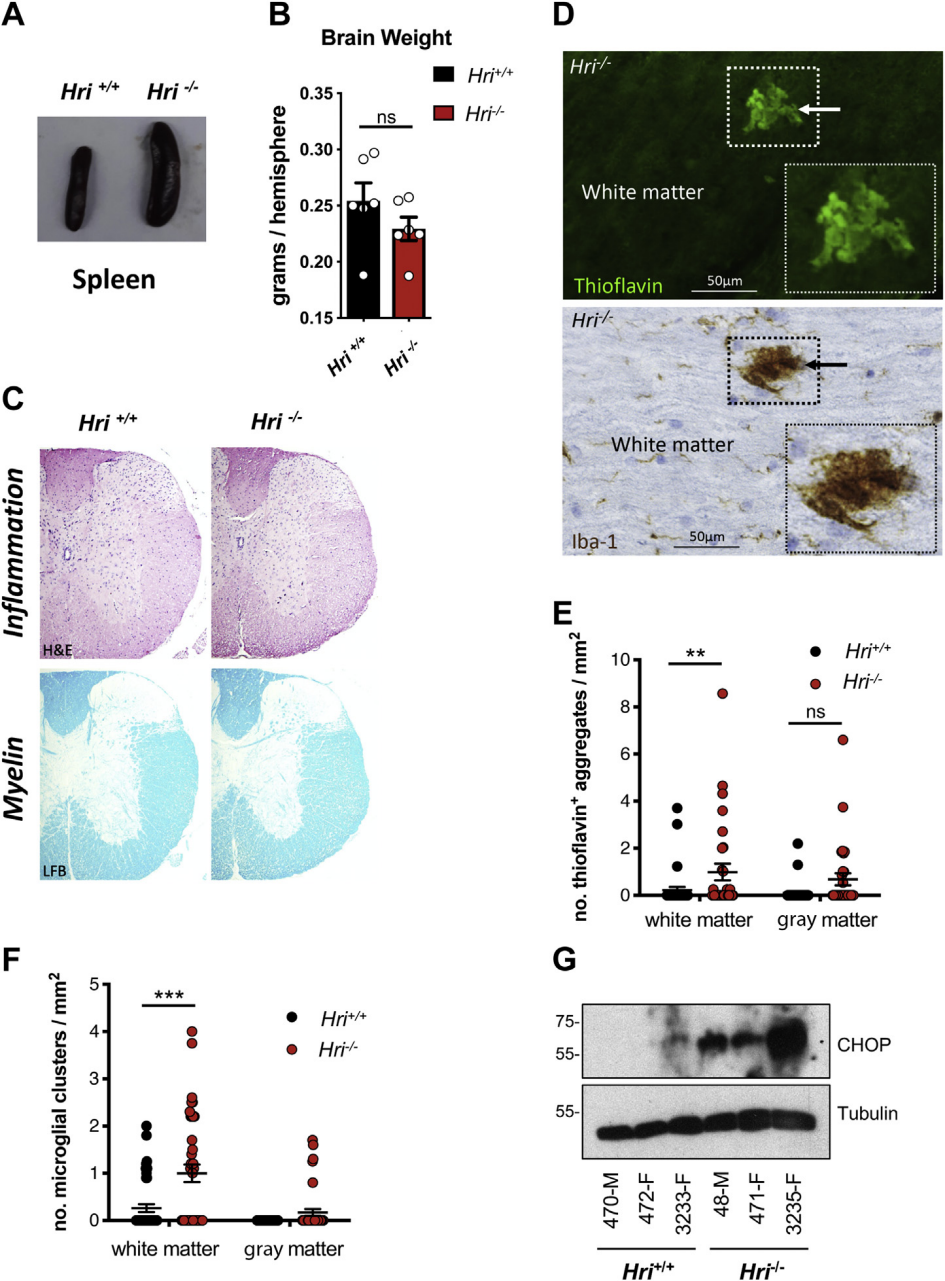
**Accumulation of protein aggregates in the central nervous system of aged *Hri***^***−/−***^**mice.***A*, splenomegaly in 7-month-old *Hri*^*−/−*^ as compared with *Hri*^*+/+*^ littermates. *B*, brain weight comparison between *Hri*^*−/−*^ as compared with *Hri*^*+/+*^ littermates (N = 6 mice per group). *C*, representative images of cross sections of spinal cord stained for H&E to visualize inflammation and LFB to visualize myelin in *Hri*^*−/−*^ as compared with *Hri*^*+/+*^ littermates. *D*, representative images of longitudinal sections of spinal cord white matter stained for Thioflavin S, to detect protein aggregates, or Iba-1, to detect microglia/macrophages. Images were from two adjacent sections, so that the same aggregate could be observed using both stains. The arrows and zoom indicate Thioflavin+ aggregates and a cluster of microglia/macrophages in the case of the Iba-1+ staining. The scale bar represents 50 μm. *E*–*F*, quantification of Thioflavin S^+^ (*E*) and Iba-1^+^ clusters (*F*) of the spinal cord white matter and gray matter from 7-month-old wildtype (*Hri*^*+/+*^) and HRI knockout (*Hri*^*−/−*^) mice (N = 6 mice per group). *G*, whole-brain lysates from 7-month-old *Hri*^*−/−*^ and *Hri*^*+/+*^ littermate mice (N = 3 mice per group; M, male; F, female) analyzed by Western blotting using the indicated antibodies. For *E* and *F*, each dot represents the score from one spinal cord coronal section with 4 to 7 sections being scored per mouse. ∗, ∗∗, and ∗∗∗ indicate *p* < 0.05, 0.01, and 0.001, respectively. Ns, not significant.

In order to gain insights into the potential role of HRI in the control of protein aggregate clearance in a more physiological setting, we sought to determine if *Hri*^*−/−*^ mice would show signs of defective clearance in the central nervous system. To this end, we kept several pairs of littermate- and sex-matched *Hri*^*−/−*^ and *Hri*^*+/+*^ mice obtained from *Hri*^*+/−*^ intercrosses until the age of 7 months. At sacrifice, *Hri*^*−/−*^ and *Hri*^*+/+*^ littermate mice appeared overall healthy, although we noted that all the knockout mice had developed splenomegaly (Fig. 5*A*), a characteristic that has been reported previously (32, 33) in *Hri*^*−/−*^ mice and relates to a progressive anemia and defective erythropoiesis in these mice. In contrast, brain weight was not significantly different between *Hri*^*−/−*^ and *Hri*^*+/+*^ littermate mice (Fig. 5*B*), and histology to detect myelin and inflammatory infiltrates in the brain (data not shown) and spinal cord (Fig. 5*C*) did not show evidence of demyelination or overt inflammation in the central nervous system of *Hri*^*−/−*^ mice. Interestingly, clusters of Iba-1^+^ microglia/macrophages were observed in the spinal cord of *Hri*^−/−^ mice and were positive for Thioflavin S, a stain specific for amyloid-type protein aggregates (Fig. 5*D*). Quantification analysis revealed a significant increase in the density of Thioflavin S^+^ aggregates (Fig. 5*E*) and Iba-1^+^ clusters (Fig. 5*F*) in the spinal cord white matter of 7-month-old *Hri*^*−/−*^ mice, suggesting that protein aggregates accumulate focally, resulting in local activation of the microglia. In support, we observed an upregulation of the protein CHOP in the brains of 7-month-old *Hri*^*−/−*^ mice, as seen in the shHRI cells upon proteasomal inhibition (Fig. 5*G*). Together, these results suggest that deletion of Hri results in the spontaneous accumulation of protein aggregates, associated with cellular stress and microglial activation in the central nervous system of aged *Hri*^*−/−*^ mice.

### Accumulation of α-synuclein in the central nervous system of aged *Hri*^*−/−*^ mice

**Figure 6:**
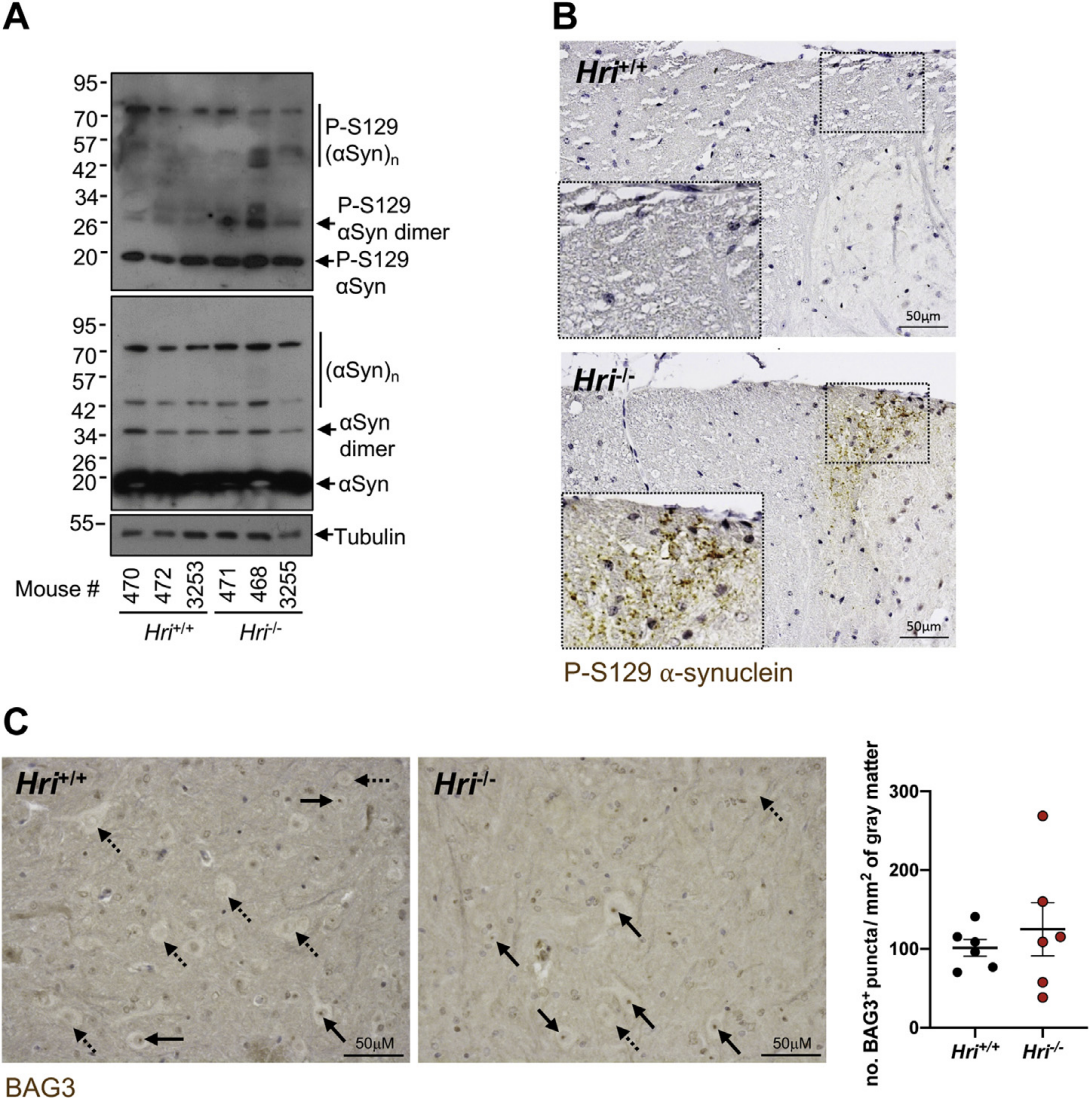
**Accumulation of α-synuclein in the central nervous system of aged *Hri***^***−/−***^**mice.***A*, whole brain lysates from 7-month-old *Hri*^*−/−*^ and *Hri*^*+/+*^ littermate mice (N = 3 mice per group) analyzed by Western blotting using the indicated antibodies. *B*–*C*, representative images of wildtype and HRI knockout mice spinal cord coronal sections stained with anti P-S129 α-synuclein antibody (*B*) and BAG3 (*C*) (N = 6 mice per group). In *C*, the *black bold arrows* indicate BAG3+ puncta in proximity of neuronal nuclei, whereas the *black dashed arrows* indicate neurons that did not show BAG3+ puncta. In *C*, dot plot represents means ± SEM of perinuclear BAG3+ puncta/mm^2^ of spinal cord gray matter from *Hri*^*−/−*^ and *Hri*^*+/+*^ littermates (N = 6 mice in each group). The scale bar represents 50 μm.

Given the results we have obtained using cellular systems, we next aimed to specifically determine if *Hri* deletion would affect α-synuclein in the mouse central nervous system. Western blot analysis of brain lysates from 7-month-old *Hri*^*−/−*^ and *Hri*^*+/+*^ littermate mice revealed accumulation of S129 phospho α-synuclein dimers and oligomers (Fig. 6*A*) in *Hri*^*−/−*^ mice. Although immunohistochemical analysis using an antibody against phospho-S129 α-synuclein did not reveal specific areas of accumulation in the brains of *Hri*^−/−^ mice as compared with *Hri*^+/+^ mice (data not shown), 5 of 6 *Hri*^−/−^ but only 2 of 6 *Hri*^+/+^ mice displayed accumulation of phospho-S129 α-synuclein in the lateral collateral pathway, a region of the sacral spinal cord horn that receives visceral sensory afferents from the bladder and distal colon (Fig. 6*B*). Finally, we investigated whether this accumulation of phospho-S129 α-synuclein was linked with defective BAG3-HSPB8–dependent CASA pathway. Immunohistological staining for BAG3 revealed BAG3+ puncta near the nucleus of a subset of neurons in the spinal cord gray matter of both *Hri*^*−/−*^ mice and *Hri*^*+/+*^ littermates controls (Fig. 6*C*). Although quantification analysis of the BAG3+ staining showed that 2 of the 6 *Hri*^*−/−*^ mice had a substantial (∼2-fold) higher number of BAG3+ puncta compared with their *Hri*^*+/+*^ littermate controls, we were likely underpowered to detect any significant differences between groups, and further studies are required to determine if defective CASA-mediated protein clearance occurs in the neurons of *Hri*^*−/−*^ mice. Together, these results suggest that *Hri* deficiency may lead to spontaneous accumulation of α-synuclein aggregates in the central nervous system in aged mice, specifically along the lateral collateral pathway that is known to be impaired in α-synucleinopathies in humans (34), supporting the notion that HRI-dependent pathways contribute to the clearance of potentially cytotoxic protein aggregates.

## Discussion

The ISR is an evolutionary conserved stress response pathway that specifically controls the protein translation machinery *via* the targeting and phosphorylation of the translation initiation factor eIF2α. It is thus not surprising that the stresses detected by the ISR relate to protein homeostasis, such as amino acid starvation or endoplasmic reticulum stress caused by protein misfolding in this organelle. In this context, it is also not unexpected that HRI, one of the four mammalian kinases of the ISR, would participate in general protein homeostasis. Our previous work had reported a key role for HRI in the control of a cytosolic unfolded protein response, or cUPR, specifically following engagement of innate immune receptors that form large molecular platforms (11). Now, the present study shows that HRI is critical for cytoprotection against proteotoxicity in cellular models of proteasome inhibition, likely by enhancing the autophagic degradation of protein cargos. In line with these observations, overexpression of α-synuclein was cytotoxic in HRI-silenced cells. In agreement, we also found that aged *Hri*^*−/−*^ mice displayed accumulation of protein aggregates and phospho-S129 α-synuclein in the central nervous system. We suggest that HRI plays a more general role in protein homeostasis by regulating the clearance of protein aggregates, likely through the upregulation of autophagy-dependent processes.

**Figure 7:**
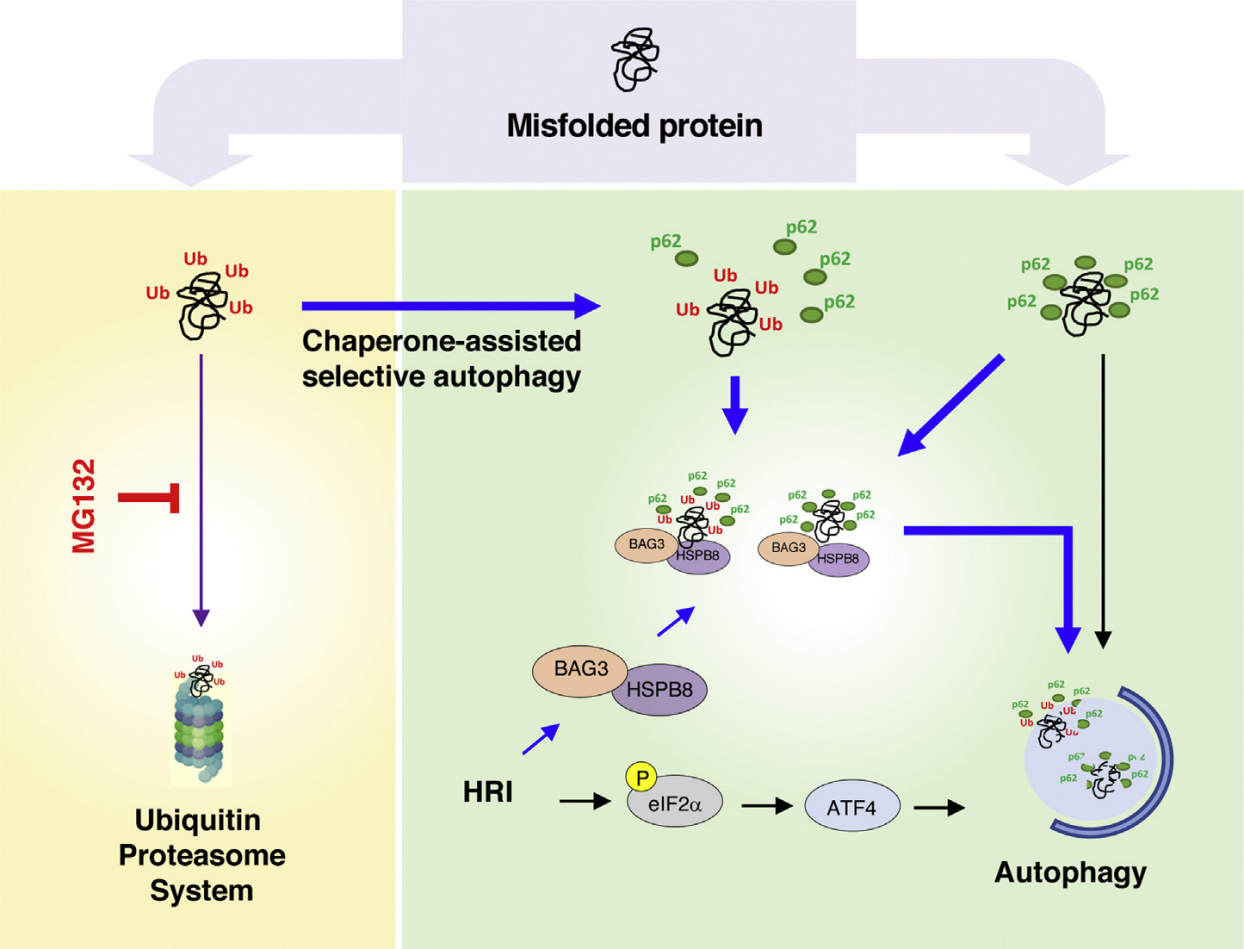
**A schematic model representing the pathway(s) modulated by HRI that maintains cellular proteostasis**. At steady state or upon cellular stress conditions, misfolded proteins are normally targeted for degradation by the ubiquitin-proteosome system (*purple line*) or lysosomal-dependent autophagy (*black line*). However, during proteosomal insufficiency induced upon MG132 treatment (*in red*), HRI-mediated chaperone-assisted selective autophagy (*blue lines*) that facilitates clearance of p62+/Ub+ as well as p62+/Ub− misfolded proteins (aggresomes) is triggered.

Previous studies have documented that the ISR drives autophagy by eIF2α kinase-dependent signaling pathways during starvation or infection (19, 35). Likewise, phosphorylation of eIF2α in response to proteasome inhibition in fibroblastic cell lines and multiple myeloma cells with either 26S proteasome inhibitors, MG132, or bortezomib is mediated by HRI (10, 17), and lack of HRI leads to impaired ATF4 signaling, which was previously shown to regulate autophagic flux downstream of eIF2α kinase (18). Thus, these lines of evidence all point to a potential role for the HRI-eIF2α-ATF4 signaling axis in the control of autophagy; however, the mechanism that connects HRI-dependent pathways to an efficient turnover of protein aggregates through autophagy during proteosomal insufficiency remains to be fully characterized. Here, we have shown that HRI contributes to the clearance of protein cargos through autophagy (see model in Fig. 7) and that blunted autophagy in HRI-silenced cells was associated with a reduction in the levels of p62, HSPB8, and BAG3, three proteins playing key roles in protein clearance by autophagy. Although the reduced levels of p62 can be at least in part associated with reduced expression of the p62 gene *SQSTM1* in shHRI cells, reduced levels of BAG3 and HSPB8 proteins in shHRI cells are caused by posttranscriptional events that remain to be characterized. It is possible that the CASA pathway is overused and strained in shHRI cells, which would result in BAG3 and HSPB8 proteins to be consumed.

Interestingly, the CASA-dependent pathway autophagy is specifically crucial for the removal of p62-positive and ubiquitin-negative protein cargos (36), which could contribute to explain why p62-positive and ubiquitin-negative aggresomes accumulated in HRI-silenced cells in proteasome-inhibited cells (see Fig. 1). Based on our previous work, which demonstrated a key role for the heat shock protein (HSP) HSPB8 in HRI-dependent control of innate immune receptor scaffolds (11), it is conceivable that similar mechanisms involving HSPs contribute to the effects observed here. For instance, it is possible that the displacement of HSPs from HRI to cytosolic protein aggregates contributes to the activation of HRI, as previously suggested in the case of Hsc70 and Hsp90 (32, 37, 38, 39, 40). Then, ISR-dependent transcriptional reprogramming could result in the upregulation of specific HSPs that help in the protein refolding and autophagic clearance of protein cargos through the CASA pathway. In this model, the impact of HRI on autophagy pathways would be twofold: (i) by regulating the expression of autophagy genes as we observed in this study, likely through the eiF2α-ATF4 axis, and (ii) by orchestrating the function of specific HSP proteins involved in CASA (Fig. 7). Further work is required to delineate the mechanisms underlying the various roles played by HRI in the control of autophagy. Nonetheless, this may have far-reaching implications in neurodegenerative disorders since autophagy, and more specifically BAG3-HSPB8–dependent CASA, has been shown to play important homeostatic motoneuron functions in the brain (41, 42).

The pathological analysis of the spinal cord from 7-month-old *Hri*^*−/−*^ mice showed deposits of protein aggregates and clusters of microglia, reminiscent of pathological features found in the central nervous system of patients with neurodegenerative diseases (43). In addition, we found that in the 7-month-old *Hri*^*−/−*^ mice α-synuclein accumulates in the lateral collateral pathway of the sacral spinal dorsal horn. This is a specialized region of the sacral spinal cord that receives visceral sensory afferents from the bladder and distal colon *via* the pelvic nerve (44). It is neurochemically distinct from other parts of the dorsal horn (45, 46) and is well conserved among mammals (44, 47). Notably, a pathological study on postmortem spinal cord tissue has previously shown that α-synuclein is deposited at this region in individuals with α-synucleinopathies, including Parkinson’s disease, dementia with Lewy bodies, and multiple system atrophy, where it has been suggested to contribute to impaired micturition and/or constipation (34). Therefore, deposition of α-synuclein in the lateral collateral pathway of the sacral spinal dorsal horn of aged *Hri*^*−/−*^ mice may be relevant to disease processes occurring in α-synucleinopathies in humans. The identification of BAG3+ puncta in close association to a subset of spinal cord neurons in old mice may reflect an accumulation of misfolded proteins with aging. Further studies with larger groups of old mice will be important to study changes that may occur in *Hri*^*−/−*^ compared with *Hri*^*+/+*^ littermates. Our data provide encouraging preliminary evidence that HRI-dependent control of the ISR may play a key homeostatic function in the central nervous system. It must be noted that these results were obtained without crossing our mice with transgenic animals that are typically used in neurodegenerative studies or using neurotoxic drugs, which can result in severe phenotypes but may be less physiologically relevant. Based on our results, it would be interesting to age *Hri*^*−/−*^ more than 7 months (up to 12–16 months) and also to perform behavioral studies to evaluate motor and cognitive impairment in these mice. With regards to the PD phenotype, a neuropathological analysis would be interesting to perform, focusing on sections of the spinal cord, brainstem (foremost the substantia nigra pars compacta of the midbrain *versus* the adjacent ventral tegmental area [an area of dopamine cells less vulnerable to PD]), hippocampus, and cortex, as well as the peripheral nervous system, which may be the initial site of onset of disease. Although these assays are outside of the scope of the present initial study, we believe that our results provide an encouraging foundation for more in-depth studies in *Hri*^*−/−*^ mice.

Importantly, our results suggest that the HRI-dependent pathway might represent a promising avenue for the treatment of neurodegenerative diseases or any pathology involving accumulation of cytotoxic protein aggregates. This approach might be more promising than targeting the more downstream event of eIF2α phosphorylation, which would have more dramatic side effects as it serves as a general hub for the multiple stresses of the ISR. Because the ISR and phosphorylation of eIF2α can be chronically upregulated in neurodegenerative diseases, attempts have been made to mitigate this pathway therapeutically (48). However, this may represent an overly simplistic approach that neglects the fact that, if stress pathways are activated, it is because an underlying condition causes the stress in the first place. Moreover, targeting the messenger would not only be ineffective but could increase pathology, given that these pathways are induced to dampen the initial stress. Based on our results, we suggest that therapeutic interventions that would aim to transiently and locally amplify HRI-dependent signaling could be a viable option against the toxicity induced by cytosolic protein aggregates. A better understanding, at the biochemical level, of the cellular processes and pathways controlled by HRI will be beneficial for the rationale design of such therapeutic strategies.

## Experimental procedures

### Cell lines and reagents

The human epithelial HeLa and HEK293T cell lines (American Type Culture Collection) were cultured in Dulbecco’s modified Eagle medium supplemented with 10% fetal calf serum, 2 mM L-glutamine, 50 IU penicillin, and 50 mg/ml streptomycin (Wisent Bio Products). Cells were maintained in 95% air and 5% CO_2_ at 37 °C. Endotoxin-free fetal calf serum and phosphate-buffered saline (PBS) were from Wisent (Saint-Bruno-de-Montarville, Quebec, Canada).

Lentiviral knockdown of HRI expression was performed as described (11), and *HRI*^*−/−*^ cell line generation and characterization were also described (11). Stimulation with tumor necrosis factor (10 ng/ml, Cell Signaling Technology) plus cycloheximide (10 μg/ml) was performed as described (49). MG132 and chloroquine were from Sigma.

### Mice

*Hri*^+/*−*^ breeder mice were obtained from a previous study (50). Mice were bred and housed under SPF conditions at the Center for Cellular and Biomolecular Research, University of Toronto. All experiments using *Hri*^*+/+*^ and *Hri*^*−/−*^ mice were performed with 7-month-old littermate mice generated from *Hri*^*+/−*^ × *Hri*^*+/−*^ cross. All mice experiments were approved by the Animal Ethics Review Committee of the University of Toronto. Genotyping was performed using primers described in a previous publication (50).

### Tissue collection

Mice whose spinal columns were harvested for histology were euthanized with CO_2_ and intracardially perfused with PBS, using a peristaltic pump. The spinal columns and brains were excised and post-fixed in 10% buffered formalin for 1 week prior to being processed into paraffin.

### Histology and immunohistochemistry

Seven-micrometer paraffin coronal sections of mouse spinal cord and brain were mounted on Superfrost Plus glass slides (Knittel Glass, Germany) and dried in the oven (Precision Compact Oven, Thermo Scientific) overnight at 37 °C. Paraffin sections were deparaffinated in xylene and rehydrated through a series of ethyl alcohol solutions.

Histology was performed using standard hematoxylin & eosin (H&E) and Luxol fast blue (LFB) stains to visualize inflammation and demyelination, respectively. The sections were subsequently dehydrated through a series of ethyl alcohol solutions and then placed in xylene before being coverslipped with Entellan mounting media (Merck Millipore, USA).

For the detection of protein aggregates, deparaffinated and rehydrated spinal cord sections were incubated in 1% Thioflavin S solution (Sigma-Aldrich, USA) for 10 min at RT. The sections were subsequently dehydrated through a series of ethyl alcohol solutions and then placed in xylene before being coverslipped with Entellan mounting media. Thioflavin S–bound protein aggregates were visualized under the microscope (Axio Imager Z1, Zeiss), using the EGFP filter (wavelength, 488 nm). Brain sections from donors with Alzheimer’s disease were used as positive controls.

For the immunohistochemistry, deparaffinated and rehydrated spinal cord sections were incubated in 0.3% H_2_O_2_ in methanol for 20 min to block endogenous peroxidase activity. Epitopes were exposed by heat-induced antigen retrieval in 10 mM sodium citrate buffer (pH 6.0) in a pressure cooker placed inside a microwave set at high power (∼800 W) for 15 min. The nonspecific binding of antibodies was blocked using 10% Normal Goat Serum (DAKO, Glostrup, Denmark) in PBS for 20 min at RT. Primary antibodies to detect either microglia/macrophages (rabbit monoclonal anti-Iba-1, clone EPR16589, abcam178847) or alpha synuclein phosphorylated on Ser129 (rabbit monoclonal anti-alpha-synuclein phospho-S129, clone EP1536Y, Abcam ab51253) were diluted (1:100) in Normal Antibody Diluent (Immunologic, Duiven, the Netherlands). Sections were incubated in the primary antibody solution overnight at 4 °C. The following day, sections were incubated with the Post Antibody Blocking Solution for monoclonal antibodies (Immunologic) diluted 1:1 in PBS for 15 min at RT. Detection was performed by incubating the sections in the secondary Poly-HRP (horseradish peroxidase)-goat anti-mouse/rabbit/rat IgG (Immunologic) antibodies diluted 1:1 in PBS for 30 min at RT followed by incubation in DAB (3,3-diaminobenzidine tetrahydrochloride; Vector Laboratories, Burlingame, CA, USA) as chromogen. Counterstaining was performed with hematoxylin (Sigma-Aldrich) for 10 min. The sections were subsequently dehydrated through a series of ethyl alcohol solutions and then placed in xylene before being coverslipped with Entellan mounting media. Sections stained with secondary antibody alone were included as negative controls.

### Image analysis and quantification

For all staining, four to seven spinal cord sections from each of six *Hri*^*+/+*^ and six *Hri*^*−/−*^ mice were scored. The H&E and LFB stains of brain and spinal cord sections were screened for evidence of inflammation or demyelination, respectively, using a light microscope (Axioscope, Zeiss) connected to a digital camera (AxioCam MRc, Zeiss) and the Zen pro 2.0 imaging software (Zeiss). Since no signs of inflammation or demyelination were observed, no further quantification analysis was performed. The Iba-1 immunostaining of brain and spinal cord sections was screened for evidence of microglial/macrophages clusters, as described (51), using a light microscope (Axioscope, Zeiss) connected to a digital camera (AxioCam MRc, Zeiss) and the Zen pro 2.0 imaging software (Zeiss). Since microglial/macrophages clusters were observed in the spinal cord but not in the brain, no further quantification analysis of Iba-1 immunostaining in the brain was performed.

The Thioflavin S stain of brain and spinal cord sections was screened for evidence of Thioflavin S–bound protein aggregates, using the EGFP filter (wavelength, 488 nm) in a microscope (Axio Imager Z1, Zeiss) connected to a digital camera (AxioCam 506 mono, Zeiss) and the Zen pro 2.0 imaging software (Zeiss). Since Thioflavin S–bound protein aggregates were observed in the spinal cord but not in the brain, no further quantification analysis of Thioflavin S immunostaining in the brain was performed.

Quantitative analysis of the Iba-1 immunostaining and the Thioflavin S staining of spinal cord sections was performed on 4× and 20× magnification, using ImageJ 1.15s (National Institute of Health, USA) imaging software. Images were calibrated in ImageJ. The white or gray matter areas of the spinal cord were measured at 4× magnification using the ImageJ freehand selections tool. The number of microglial/macrophages clusters and the number of Thioflavin S–bound protein aggregates was counted at 20× magnification over the entire white matter or gray matter areas of the spinal cord. Staining is expressed as number of microglial/macrophages clusters or the number of Thioflavin S–bound protein aggregates per square millimeter of white matter or gray matter areas.

### Western blots

Unless otherwise indicated cells were lysed using RIPA buffer supplemented with protease and phosphatase inhibitors on ice. Lysates were then centrifuged at 13,000*g* to separate out membrane fractions/insoluble fractions and cytoplasmic proteins/soluble fractions. The membrane fractions were resuspended in RIPA buffer with Laemmli blue loading buffer and boiled to obtain the insoluble fractions. Samples were then run on acrylamide gels and transferred onto PVDF for blotting. For blotting of aggregates, membranes were first fixed for 30 min with 0.4% paraformaldehyde. Membranes were then blocked with 5% milk or BSA in Tris-Buffer Saline with Tween and incubated with the indicated antibodies: mouse monoclonal anti-Tubulin (#T5168, Sigma, 1:10,000 dilution), rabbit anti-cleaved PARP (#9546, Cell Signaling Technology, 1/1000), LC3-A/B (#4108, Cell Signaling Technology), rabbit anti-GFP (#ab290, Abcam, 1:5000), rabbit anti-Hsc70 (#ab51052, Abcam, 1:1000), mouse anti-α-synuclein (#ab1903, Abcam 1/3000), rabbit anti-α-synuclein phospho-S129 (#ab51253, Abcam 1:3000), Anti-SQSTM1/p62 antibody (#ab56416, Abcam 1:5000), Hsp22/HSPB8 (ab151552, Abcam 1:1000), Anti-DDIT3 antibody [9C8] (#ab11419, Abcam 1:1000), BAG3 antibody (#NBP2-27398, Novus 1/2500), BAG3 antibody (#NBP1-86442, Novus 1:2500), Anti-Atg16L polyclonal antibody (#PM040, MBL, 1:5000), Anti-Ubiquitin Mouse mAb (FK2) (ST1200, Millipore, 1:1000).

### Coimmunoprecipitation

For coimmunoprecipitation experiments cells were lysed using RIPA buffer supplemented with protease and phosphatase inhibitors on ice and subsequently centrifuged at 13,000*g* for 3 min. Protein G beads (Pierce) were washed twice with RIPA buffer and centrifuged at 2000*g* for 10 min before adding lysate and the indicated antibodies. The immunoprecipitation reaction was incubated overnight at 4 °C. The following day samples were centrifuged at 10,000*g* for 5 min and washed three times with RIPA buffer supplemented with protease and phosphatase inhibitors before finally being resuspended in 120 μl of RIPA buffer with Laemmli blue loading buffer and boiled for 10 min before samples were subjected to SDS-PAGE.

### Immunofluorescence

HeLa cells were seeded onto glass coverslips (Warner Instruments) placed in 24-well plates at a density of 1.2 x 10^5^. Following stimulation cells were fixed either with 4% paraformaldehyde for 10 min or with ice-cold 100% methanol for 5 min. Coverslips were then washed twice with PBS and then permeabilized with 0.1% Triton in PBS for 10 min at room temperature. Samples were then washed two times with PBS and blocked for half an hour with blocking buffer (1% BSA in PBS). Coverslips were then incubated with the indicated antibodies for 30 min in blocking buffer and washed twice with PBS in between antibody incubations. Antibodies were used as follows: Ubiquitin, (1:200, Millipore), p62 (1:200, Novus), p62 (1:250, Abcam), BAG3 (1:250, Novus), Atg16L (1:500, MBL). Before mounting cells were stained with DAPI (4’,6-diamidino-2-phenylindole) dye (Thermo Fisher) and mounted with Dako mounting medium and imaged by confocal microscopy as described (52).

### RNA isolation and quantitative RT-PCR

RNA extraction from cells was carried out using the GeneJET RNA purification kit (Thermo Fisher Scientific), and subsequently, eluted RNA was amplified according to Verso cDNA synthesis kit (Thermo Fisher Scientific) according to the manufacturers’ protocol. cDNA was prepared from 1 μg of total RNA using oligo(dT), dNTP, RNase Enhancer, and Moloney murine leukemia virus reverse transcriptase. cDNA was diluted accordingly and prepared in 10-μl reactions using PowerUp SYBR Green Mastermix (Applied Biosystems). The CFX384 Touch real-time PCR detection system (Bio-Rad) was used to obtain the raw *C*_*t*_ values. Fold change in gene expression was calculated by 2^−ΔΔ*Ct*^ formula. Primer pair sequences used for quantitative real-time PCR (qRT-PCR) analysis are as follows: human *p62* (Forward) 5′-AAGCCGGGTGGGAATGTTG-3′ and human *p62* (Reverse) 5′-GCTTGGCCCTTCGGATTCT-3′; Human *ATF4* (Forward) 5′-CCAACAACAGCAAGGAGGAT-3′ and human *ATF4* (Reverse) 5′-GGGGCAAAGAGATCACAAGT-3′; *ATG16L1* (Forward) 5′-AACGCTGTGCAGTTCAGTC-3′ and human *ATG16L1* (Reverse) 5′-AGCTGCTAAGAGGTAAGATCCA-3′; human *MAP1LC3A* (Forward) 5′-AACATGAGCGAGTTGGTCAAG-3′ and human *MAP1LC3A* (Reverse) 5′-GCTCGTAGATGTCCGCGAT-3′; human *TBP* (Forward) 5′-GGGCATTATTTGTGCACTGAGA-3′ and human *TBP* (Reverse) 5′-TAGCAGCACGGTATGAGCAACT-3′; human *BAG3* (Forward) 5′-AGCTCCGACCAGGCTACATT-3′ and human *BAG3* (Reverse) 5′-GGATAGACATGGAAAGGGTGC-3′; human *HSPB8* (Forward) 5′-AAAGATGGATACGTGGAGGTG-3′ and human *HSPB8* (Reverse) 5′-GGGAAAGTGAGGCAAATACTG-3′.

### Transfections

For transient expression, transfections were performed with PEI MW25000, and cells were lysed after overnight transfection. Plasmid encoding αsyn-GFP was obtained from Addgene. Plasmids encoding αsyn-L1 or αsyn-L2 have been described elsewhere (29, 30).

### Luciferase assays

Luciferase assays to measure α-synuclein oligomerization were performed as described (29, 30). HEK293T cells were transiently transfected in 6-well plates with Lipofectamine 2000 (Life Technologies) as per the manufacturer’s instructions. Plasmids transfected included αsyn-L1+αsyn-L2 or syn-L1+pcDNA combinations, with 1 μg of each plasmid used in each combination. At 24 h post transfection, the supernatant (1 ml) was centrifuged at 3000*g* for 5 min at 4 °C. The cleared supernatant was used in the luciferase assay. The cells were scraped from each culture well in 1 ml PBS, and then 50 μl of cells was transferred in triplicate to a 96-well, flat-bottom, opaque white plate (Greiner). The remaining cells were used for Western blot analyses. Native coelenterazine (Prolume), a cell permeable substrate of Gaussia luciferase, was resuspended in nanofuel solvent (Prolume) to 5 mg/ml and dispensed per well to a final concentration of 20 mM by an automated plate reader (CLARIOstar, BMG Labtech). The bioluminescent signal generated by the luciferase enzyme was integrated over 2 s before measurement at 480 nm. Cotransfection of a GFP vector was used to normalize for transfection efficiency in some experiments.

### Statistics

For all experiments statistics was performed using GraphPad Prism software 8.0 (GraphPad Software Inc, San Diego, CA, USA). Results of the experiments in cell lines were analyzed by Student’s *t* test or one-way ANOVA. For quantifications of thioflavin aggregates and microglial clusters, statistics was performed by multiple *t* test analysis and results were considered significant when *p* < 0.05. Statistical significance in figure panels have been denoted as “∗”, “∗∗”, and “∗∗∗” for *p* < 0.05, *p* < 0.01, and *p* < 0.001, respectively.

## Data availability

All the data are in the manuscript.

## Conflict of interest

The authors declare that they have no conflicts of interest with the contents of this article.
