# Supplementary material for: The eIF2α kinase HRI triggers the autophagic clearance of cytosolic protein aggregates
Source: J Biol Chem. 2020 Dec 2;296:100050. doi: 10.1074/jbc.RA120.014415 (PMC7948985; doi:10.1074/jbc.RA120.014415)
Supplement: Supporting Information [file mmc1.pdf]

1C

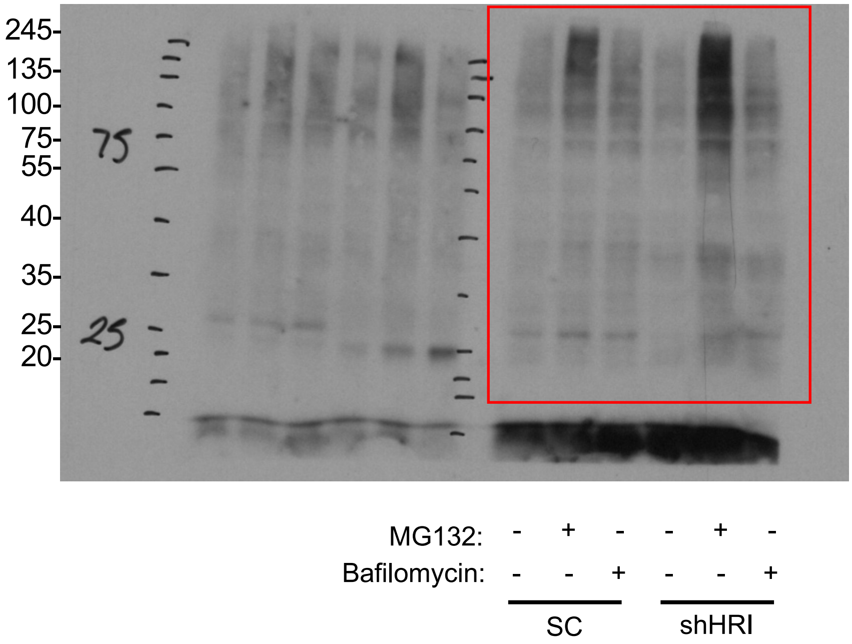

Source Data Figure 1

2A

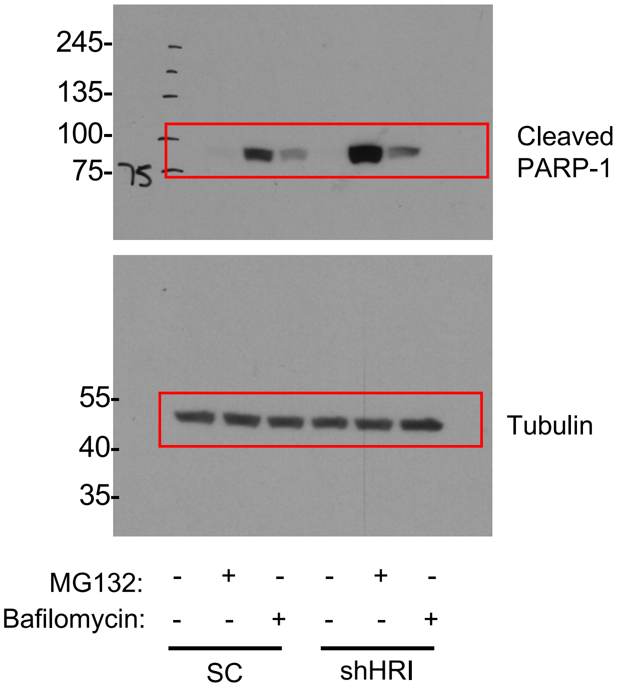

2B

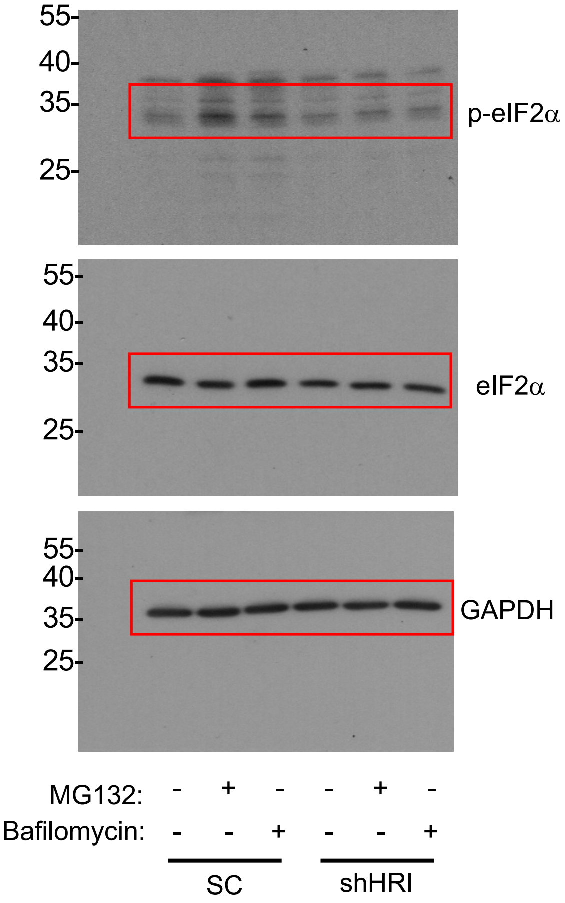

Source Data Figure 2

2C

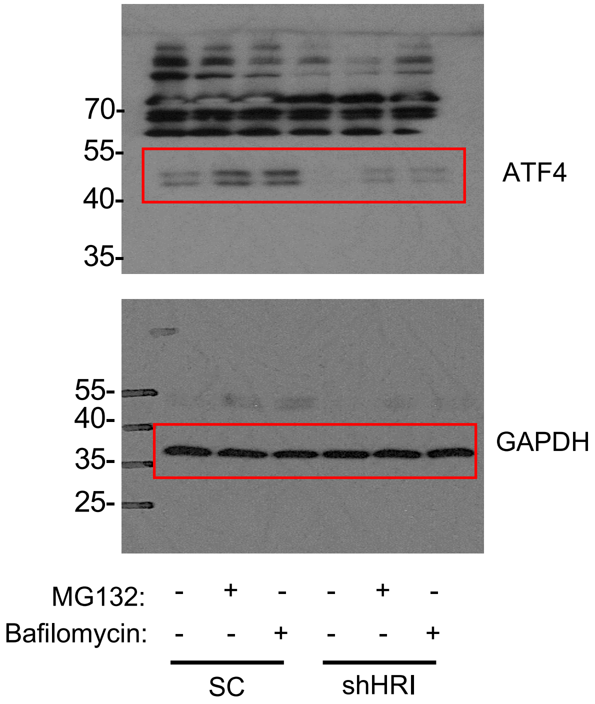

2D

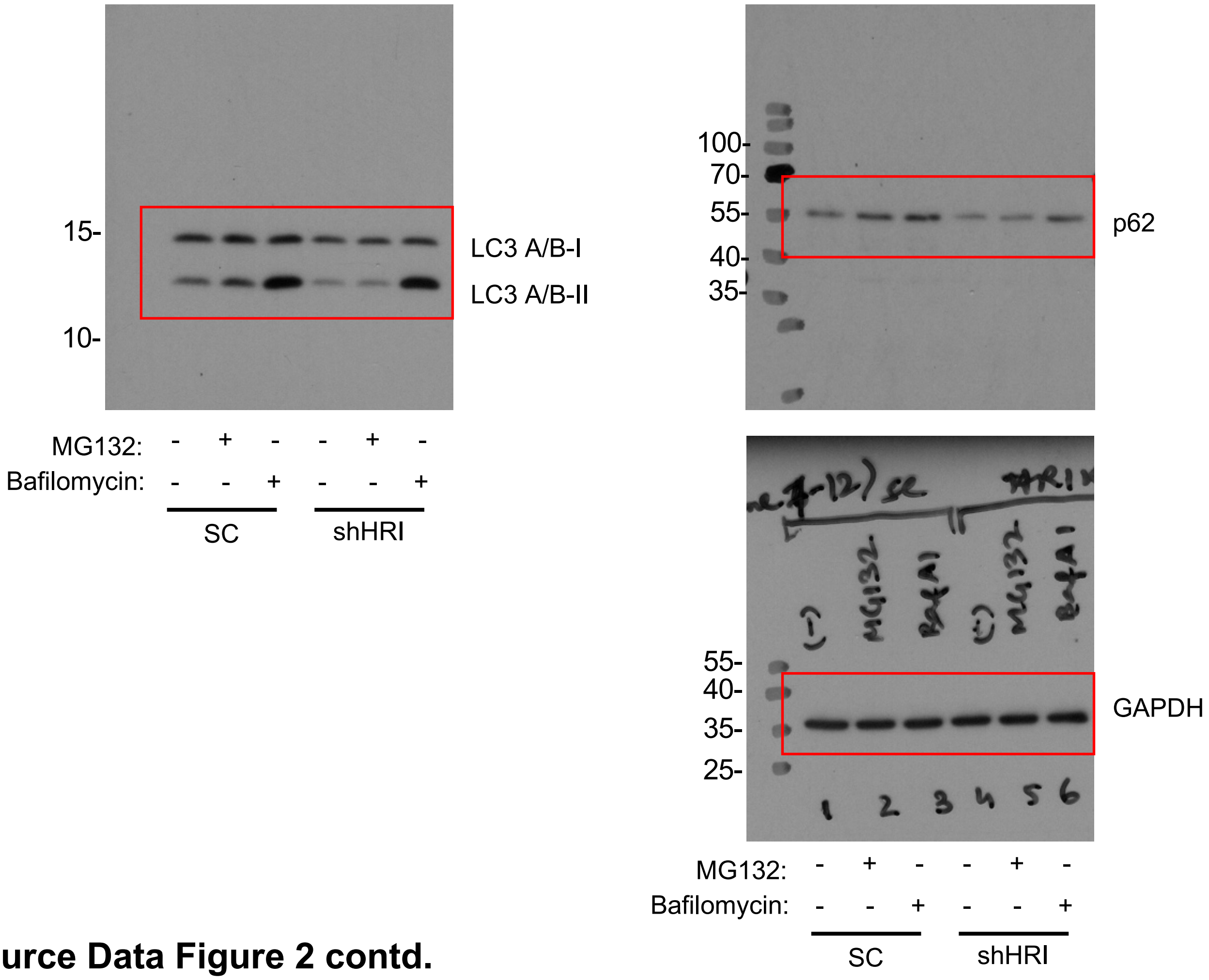

Source Data Figure 2 contd.

3A

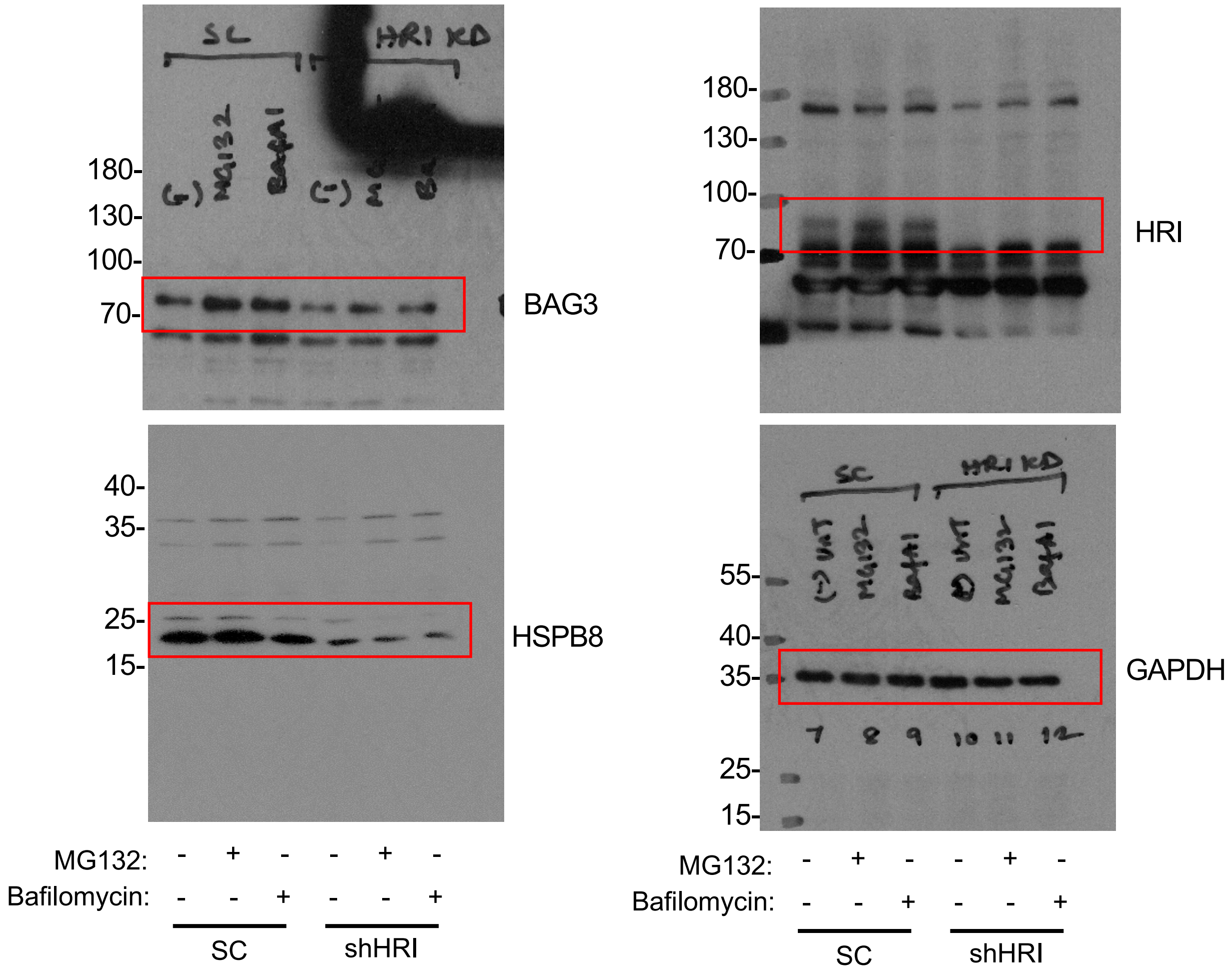

Source Data Figure 3

4B

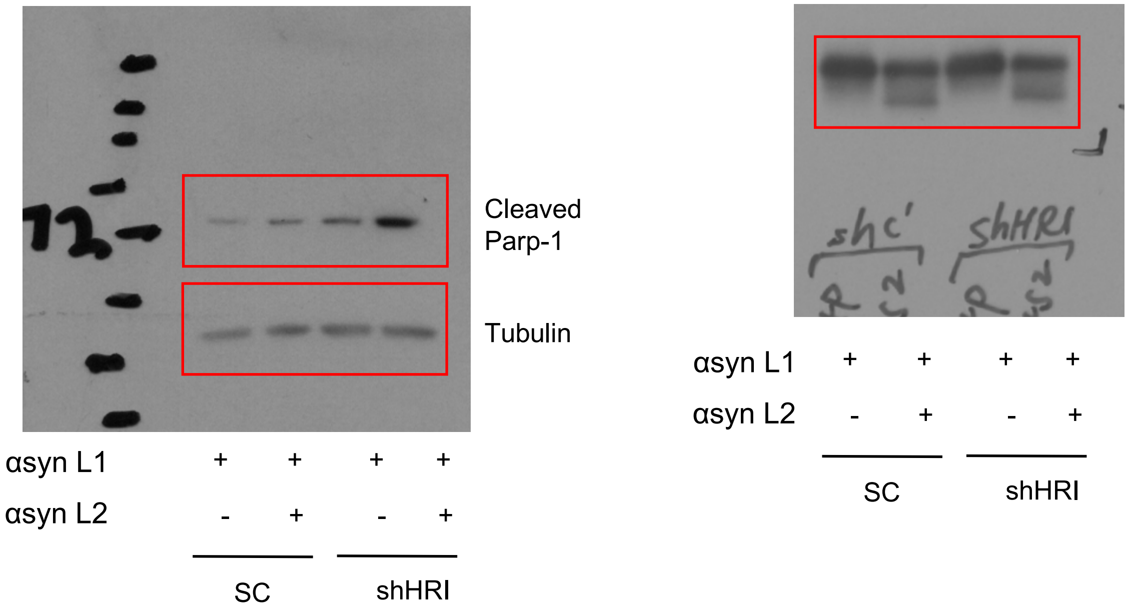

4C

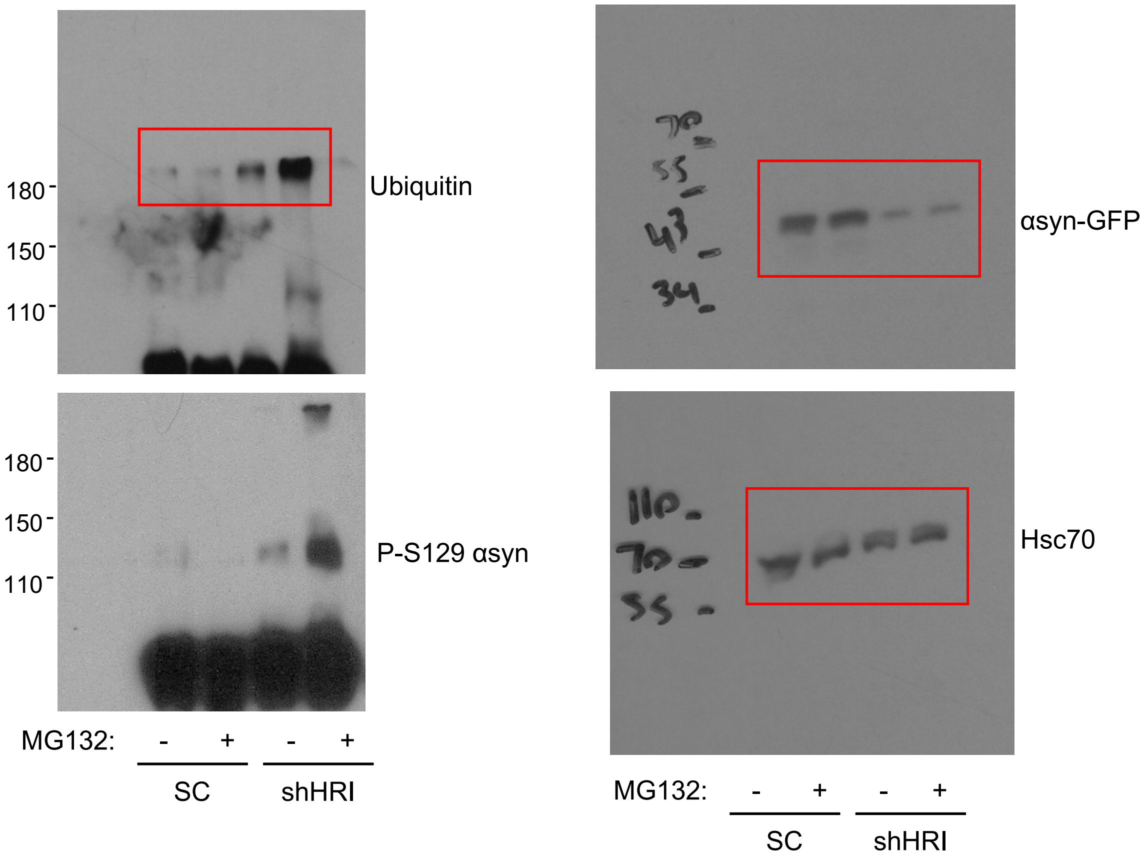

Source Data Figure 4

5G

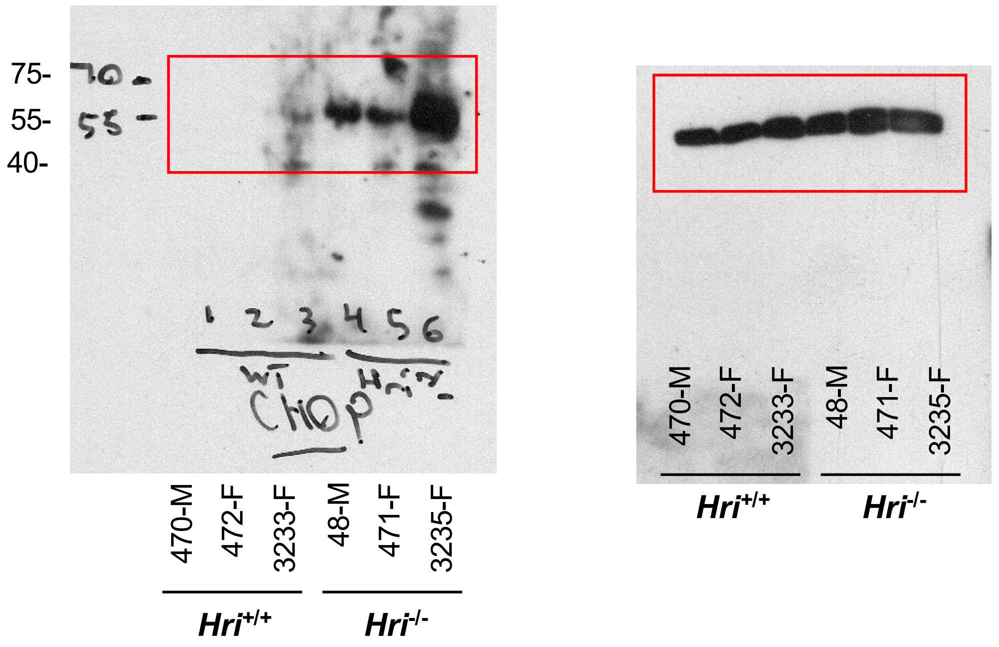

Source Data Figure 5

6A

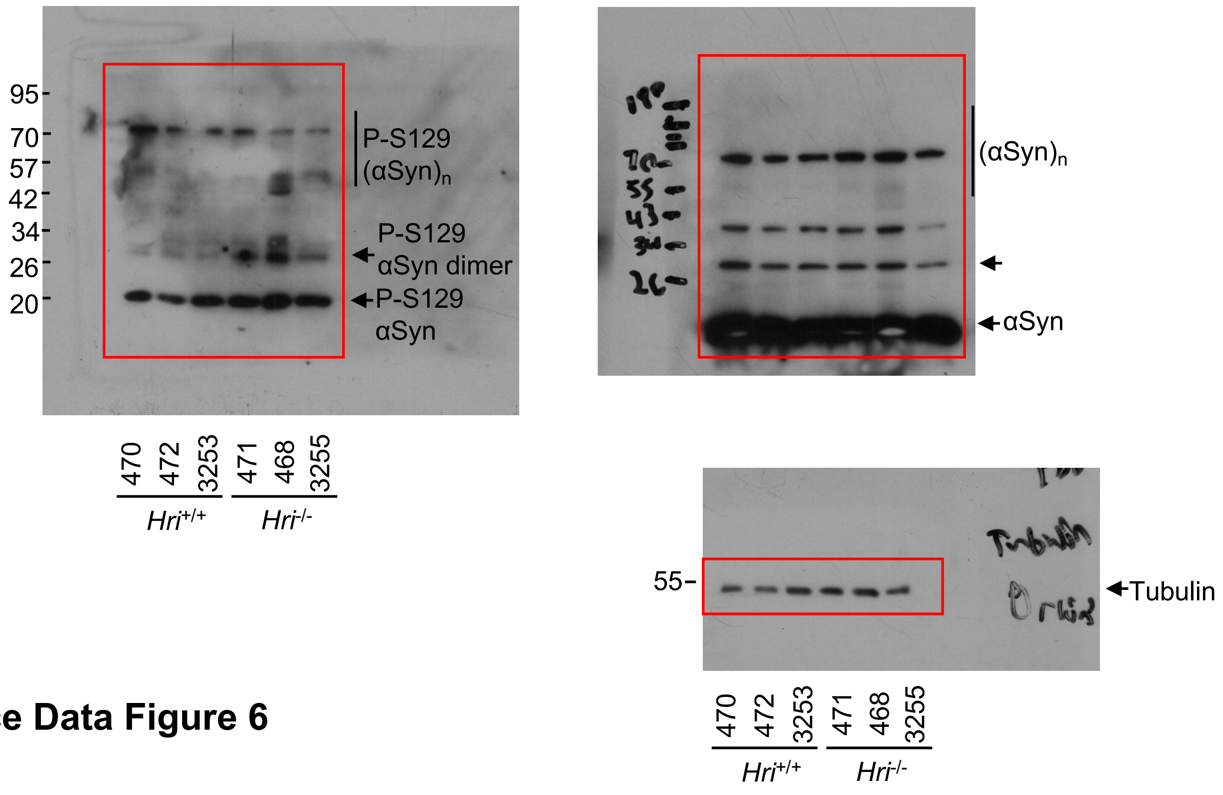

Source Data Figure 6
