# Supplementary material for: The eIF2α kinase HRI triggers the autophagic clearance of cytosolic protein aggregates
Source: J Biol Chem. 2020 Dec 2;296:100050. doi: 10.1074/jbc.RA120.014415 (PMC7948985; doi:10.1074/jbc.RA120.014415)
Supplement: Supporting Information 2 [file mmc2.pdf]

S1A

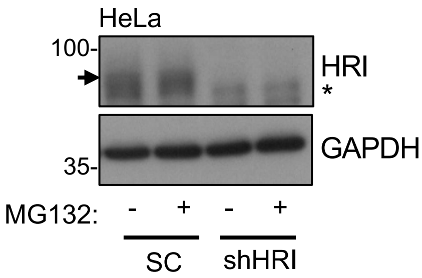

S1B

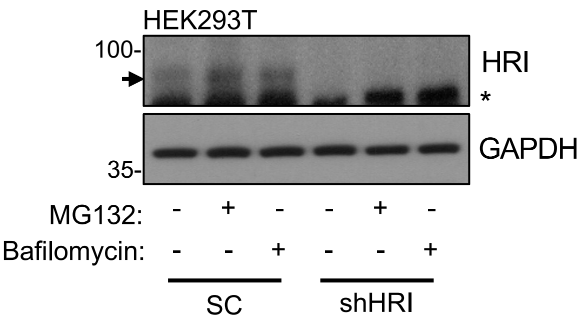

Supplementary Figure 1

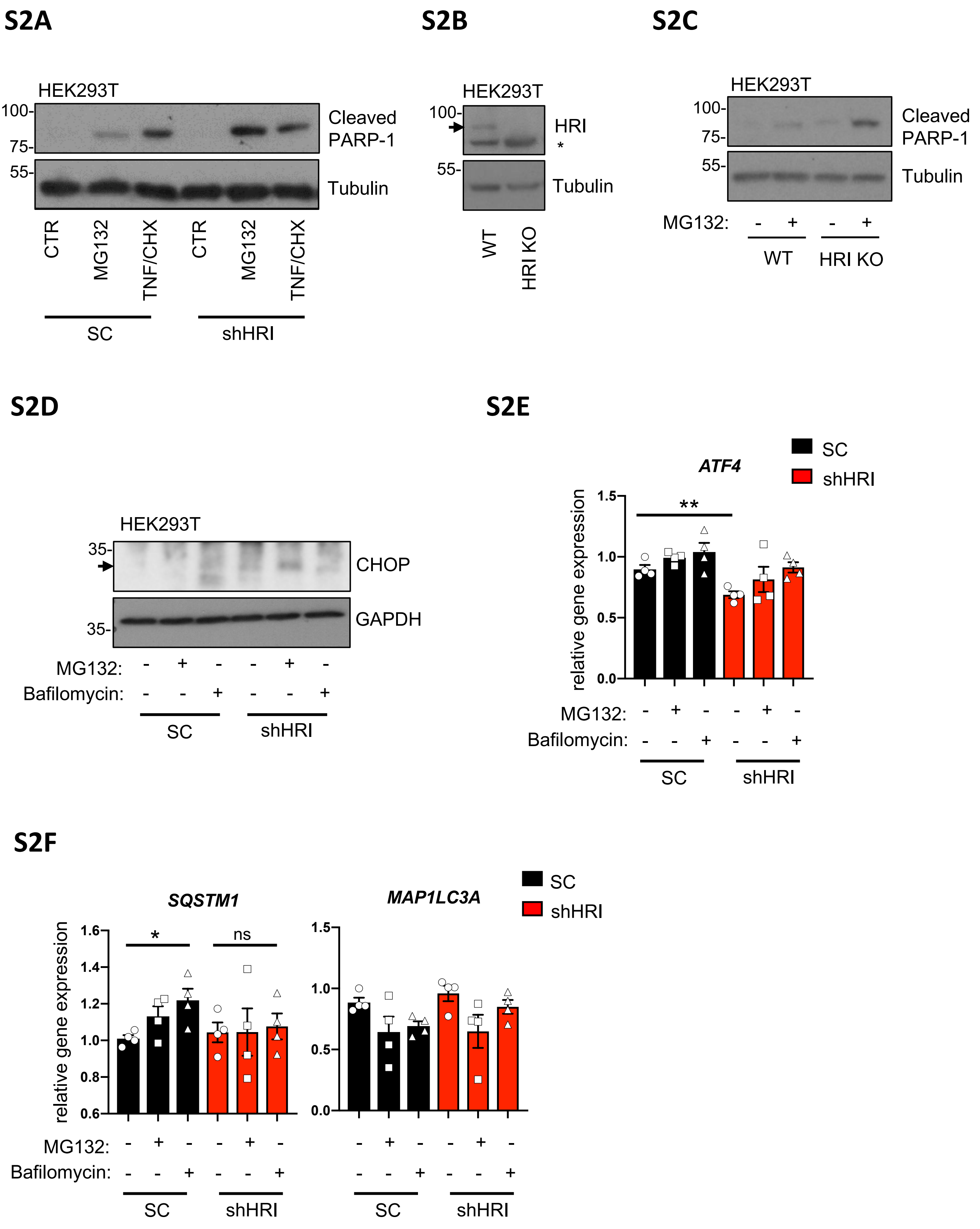

Supplementary Figure 2

S3A

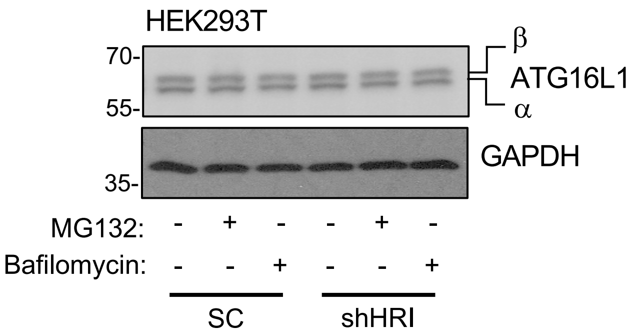

S3B

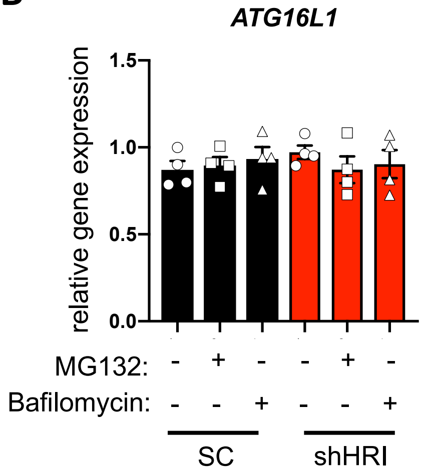

S3C

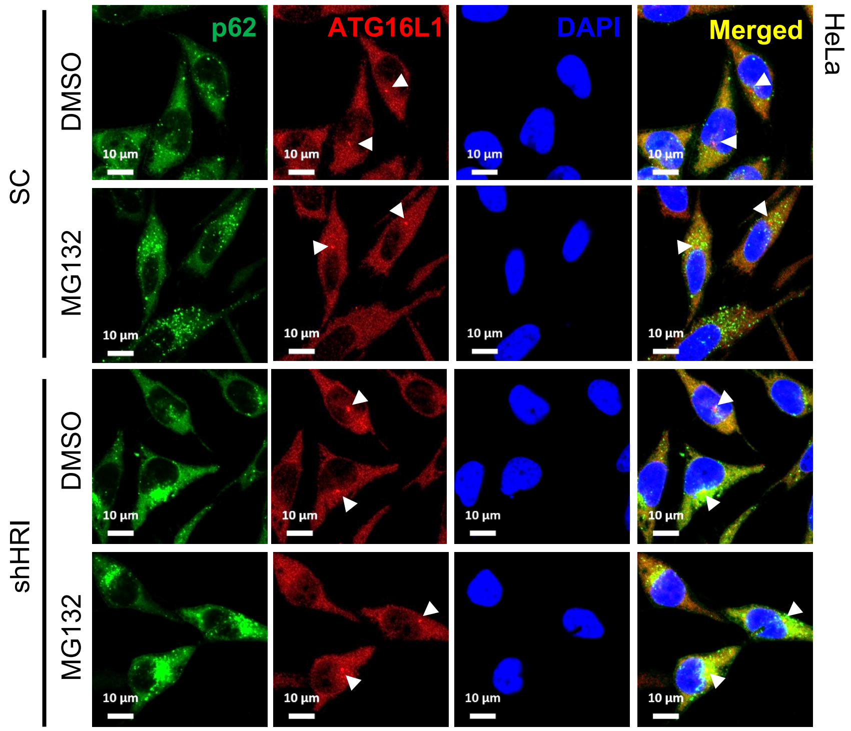

Supplementary Figure 3

SUPPLEMENTARY FIGURE LEGENDS

Supplementary Figure 1: Validation of HRI-knockdown in HeLa and HEK293T cells.

S1A-B. HeLa (A) and HEK293T (B) cells transduced with lentiviral particles, targeting either a scrambled (SC) sequence or HRI (shHRI) were treated either with DMSO (control), MG132 (5μM for HeLa and 10μM for HEK293T) or bafilomycin (10nM) for 4 hours as indicated in the figures. Subsequently, cell extracts were subjected to western blotting with antibodies against HRI. GAPDH served as a loading control. Arrow indicates specific HRI band.

Supplementary Figure 2: Impaired autophagic flux in HRI-deficient cells is associated with increased cell death.

S2A. Scrambled (SC) sequence and HRI (shHRI) HEK293T cells treated with DMSO, 10μM MG132 or TNF plus cycloheximide (CHX) for 4 hours and cell extracts were subjected to western blot analysis and probed with antibodies against cleaved PARP-1 and tubulin. **S2B.** Validation of CRISPR-Cas9 mediated HRI-knockout (HRI KO) HEK293T cells and wild-type (WT) control. Arrow indicates specific HRI band. **S2C.** Western blot analysis was carried out with cell extracts derived from WT and HRI-KO HEK293T cells treated with either DMSO or 10μM MG132 for 4 hours and blots were probed for cleaved PARP-1. Tubulin was used as loading control for this western blot analysis. **S2D.** Scrambled (SC) sequence and HRI (shHRI) HEK293T cells were treated for 4 hours with DMSO (control), 10μM MG132 and 10nM bafilomycin and cell extracts were subjected for western blot analysis and probed with CHOP antibody. Arrow indicates specific CHOP band **S2E-F.** qRT-PCR analysis of *ATF4* (E), *SQSTM1* and *MAP1LC3A* (F) in SC and shHRI HEK293T cells treated with DMSO, 10μM MG132 or 10 nM bafilomycin for 4 hours. Figure panel A-D and E-F are representative of 3 and 4 independent experiments, respectively. * and ** indicates $P < 0.05$ and 0.01. ns - not significant

Supplementary Figure 3: HRI-deficiency does not impact ATG16L1, an essential autophagy protein.

A-B. Scrambled (SC) sequence and HRI (shHRI) HEK293T cells were treated for 4 hours with DMSO (control), 10μM MG132 and 10nM bafilomycin. Cell extracts were collected were either subjected for protein analysis by western blot (A) or mRNA analysis by qRT-PCR (B) for ATG16L1. **C.** Scrambled (SC) sequence and HRI (shHRI) HeLa cells treated either with DMSO (control) or with 5μM MG132 for 4 hours, followed by fixation with ice-cold 100% Methanol for 5 mins and then were subjected to immunofluorescence analysis using anti-ATG16L and anti-p62 antibodies. Scale bar: 10μm. Figure panel A-C are representative of 3-4 independent experiments.
